# Subunits of the mechano-electrical transduction channel, Tmc1/2b, require Tmie to localize in zebrafish sensory hair cells

**DOI:** 10.1371/journal.pgen.1007635

**Published:** 2019-02-06

**Authors:** Itallia V. Pacentine, Teresa Nicolson

**Affiliations:** Oregon Hearing Research Center and Vollum Institute, Oregon Health & Science University, Portland, Oregon, United States of America; University of Washington, UNITED STATES

## Abstract

Mutations in transmembrane inner ear (*TMIE)* cause deafness in humans; previous studies suggest involvement in the mechano-electrical transduction (MET) complex in sensory hair cells, but TMIE’s precise role is unclear. In *tmie* zebrafish mutants, we observed that GFP-tagged Tmc1 and Tmc2b, which are subunits of the MET channel, fail to target to the hair bundle. In contrast, overexpression of Tmie strongly enhances the targeting of Tmc1-GFP and Tmc2b-GFP to stereocilia. To identify the motifs of Tmie underlying the regulation of the Tmcs, we systematically deleted or replaced peptide segments. We then assessed localization and functional rescue of each mutated/chimeric form of Tmie in *tmie* mutants. We determined that the first putative helix was dispensable and identified a novel critical region of Tmie, the extracellular region and transmembrane domain, which is required for both mechanosensitivity and Tmc2b-GFP expression in bundles. Collectively, our results suggest that Tmie’s role in sensory hair cells is to target and stabilize Tmc channel subunits to the site of MET.

## Author summary

Hair cells mediate hearing and balance through the activity of a mechanosensitive channel in the cell membrane. The transmembrane inner ear (TMIE) protein is an essential component of the protein complex that gates this so-called mechanotransduction channel. While it is known that loss of TMIE results in deafness, the function of TMIE within the complex is unclear. Using zebrafish as a deafness model, Pacentine and Nicolson demonstrate that Tmie is required for the localization of the channel subunits, transmembrane channel-like (Tmc) proteins Tmc1/2b. They then evaluate thirteen unique versions of Tmie, each containing mutations in different domains of Tmie. This analysis reveals that specific mutated versions of Tmie reduce hair cell activity, and that these same dysfunctional versions also cause reduced Tmc expression at the normal site of the channel. These findings link hair cell activity with the levels of Tmc in the bundle, reinforcing the notion that the Tmcs are the pore-forming subunits of the mechanotransduction channel. The authors conclude that Tmie, through distinct regions, is involved in both trafficking and stabilizing the channels at the site of mechanotransduction.

## Introduction

The auditory and vestibular systems detect mechanical stimuli such as sound, gravity, and acceleration. These two systems share a sensory cell type called hair cells. The somas of hair cells are embedded in the sensory epithelium and extend villi-like processes from their apex into the surrounding fluid. The shorter of these, the stereocilia, are arranged in a staircase-like pattern adjacent to a single primary cilium known as a kinocilium. Long proteins linkages tether neighboring cilia together. Deflection of the kinocilium along the excitatory axis tugs the interconnected stereocilia, which move as a single unit called the hair bundle [1]. When tension is placed on the upper-most linkages known as tip links, the force is thought to open mechanosensitive channels at the distal end of the shorter stereocilia [2, 3]. These channels pass current, depolarizing the cell and permitting electrical output to the brain via the eighth cranial nerve. The conversion of a mechanical stimulus into an electrical signal is known as mechano-electrical transduction (MET) [4]. Aside from the channel, several other proteins converge at the base of the tip links to form a molecular complex that gates the channel. While a handful of essential members of this MET complex have been identified, we do not fully understand how all of these proteins interact. It is also not known how the MET channel is localized to and maintained at the stereocilia tips.

To characterize the molecular underpinnings of MET and the underlying cause of pathology in human patients, it is essential to examine the individual components of the transduction complex in a comprehensive fashion. Thus far, only a few proteins have been designated as members of the MET complex, and more than one protein may comprise the channel subunits. Because the MET channel has yet to be reconstituted in a hetereologous system, the identity of the pore-forming subunits of the channel remains uncertain. However, several studies in recent years have revealed strong candidates for the pore-forming subunits: the Transmembrane Channel-like (TMC) proteins TMC1 and TMC2. Mutations in *TMC1* cause human deafness [5], and double knock-outs of mouse *Tmc1/2* result in the loss of MET currents [6–8]. In zebrafish, Tmc2a and Tmc2b are required for MET in hair cells of the lateral line organ [9], and overexpression of a fragment of Tmc2a generates a dominant negative effect on hair-cell mechanosensitivity, suggestive of direct interference with the channel [10]. The TMCs localize to the tips of stereocilia, the site of MET, in mice and zebrafish [3, 6, 8, 9, 11, 12]. In TMC2 knockout mice, permeation properties of the MET channel are altered [13]. Likewise, a point mutation in mouse *Tmc1* results in altered channel properties, suggesting direct changes to the pore [7, 14]. A recent paper used a cysteine modification assay to demonstrate that modification of certain residues lining the predicted pore of TMC1 results in changes to channel conductance [15]. Furthermore, the authors demonstrated that adding a MET channel blocker during exposure to the cysteine modifier prevented their modification, implying that the unaffected residues line the inner pore and are thus inaccessible when the channel is blocked. This body of evidence concludes that the TMCs are essential subunits of the MET channel, and are likely to at least partially constitute the pore.

Another key component of the complex is Protocadherin-15 (PCDH15), which comprises the lower end of the tip link [16, 17] and interacts with the TMCs [8, 10]. A fourth membrane protein, Lipoma HMGIC fusion partner-like 5 (LHFPL5, formerly called TMHS), interacts with PCDH15 and is critical for localizing PCDH15 to the site of MET [18, 19]. LHFPL5 is also required to properly localize TMC1 in mouse cochlear hair cells [8]. However, loss of LHFPL5 in cochlear hair cells does not completely abolish MET currents, and currents can be rescued by overexpression of PCDH15 [19]. This evidence suggests that LHFPL5 is not essential but rather acts as an accessory protein. Another TMC1/2 interacting partner is Calcium and integrin binding protein 2 (CIB2), which is a cytosolic protein that is localized in stereocilia and required for MET in cochlear hair cells [20].

A sixth essential member of the MET complex is the transmembrane inner ear (TMIE) protein. Loss of TMIE results in deafness in fish, mice and humans [21–26]. A recent study suggested that TMIE is required for mechanosensitivity in cochlear hair cells of mice [27]. These authors showed that despite normal morphology of the inner ear, hair cells lacking TMIE fail to label with aminoglycosides or FM 1–43, both of which are known to permeate the MET channel [28, 29]. TMIE was first localized to the stereocilia of hair cells [30, 31], and then to the stereocilia tips where MET occurs [26]. Zhao et al. [26] further demonstrated that loss of TMIE ablates MET currents, that TMIE interacts with both LHFPL5 and the CD2 isoform of PCDH15, and that interfering with the TMIE-CD2 interaction alters MET. They proposed that TMIE could be a force-coupler between the tip link and channel. However, the CD2 isoform of PCDH15 is only essential in cochlear hair cells and not vestibular hair cells [32]. Zebrafish do not possess the CD2 isoform [10, 33], and yet they still require Tmie for hair-cell function [22]. These findings raised the tantalizing possibility that Tmie might have an additional role in MET that is independent from the tip links. Here, we present an alternative role for Tmie in hair-cell function.

We first confirmed that mechanosensitivity is absent in a previously reported zebrafish mutant of *tmie*, *ru1000* [22], and demonstrated that this defect is rescued by transgenic Tmie-GFP. The localization of Tmie-GFP is maintained in the absence of other transduction components, suggesting that Tmie trafficks independently to hair bundles. Unexpectedly, GFP-tagged Tmcs fail to localize to the hair bundle in *tmie* mutants, and overexpression of Tmie leads to a corresponding increase in bundle expression of Tmc1 and Tmc2b. To determine which regions of Tmie are involved in regulating the Tmcs, we performed a domain analysis of Tmie by expressing mutated or chimeric transgenes of *tmie* in *tmie*^*ru1000*^ larvae, and made three key discoveries: (*i*) Tmie can function without its putative first transmembrane domain, (*ii*) the remaining helix (2TM) and adjacent regions are responsible for Tmie’s function in hair cells, and (*iii*) dysfunctional versions of Tmie have reduced efficacy in localizing the Tmcs, supporting the conclusion that impaired MET is due to reduction of Tmc protein in hair bundles. Our evidence suggests that Tmie’s role in the MET complex is to promote localization of Tmc1/2 to the site of MET in zebrafish sensory hair cells.

## Results

### Gross morphology is normal in *tmie*^*ru1000*^ mutant zebrafish

**Fig 1:**
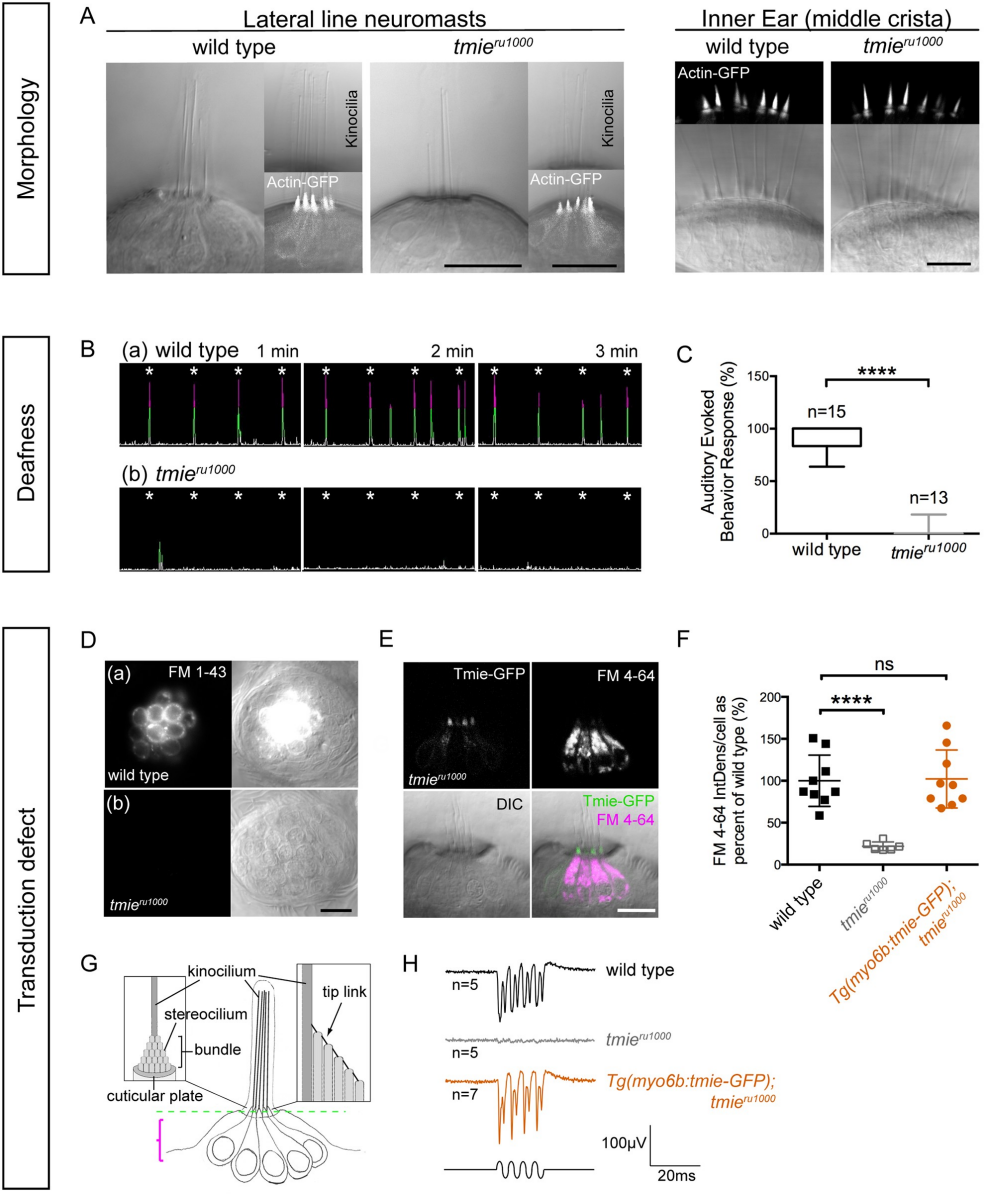
Zebrafish *tmie*^*ru1000*^ mutants: Phenotype and functional rescue by Tmie-GFP. All confocal images are of live, anesthetized larvae. (A) Hair cells in the lateral-line neuromasts (7 dpf) and inner ear cristae (5 dpf) from wild type and *tmie*^*ru1000*^ larvae. A transgene (Actin-GFP) was used to visualize stereocilia bundles. (B) Sample traces from an auditory evoked behavior response (AEBR) assay, performed on 6 dpf larvae over the course of 3 minutes. Pure tone stimuli are indicated by asterisks. Peaks represent pixel changes due to larval movements (magenta indicates positive response). (C) Quantification of AEBR displayed as box-and-whiskers plot; significance determined by two-tailed unpaired t-test with Welch’s correction. (D) Top-down view of neuromasts from 4 dpf larvae after brief exposure to a vital dye, FM 1–43. FM 1–43 and FM4-64 permeate open transduction channels. (E) Lateral view of a neuromast from a 4 dpf *tmie*^*ru1000*^ larva expressing transgenic Tmie-GFP, after exposure to FM 4–64. (F) Quantification of FM 4–64 fluorescence/cell in 5 dpf larvae; significance determined by one-way ANOVA. (G) A cartoon depiction of a group of lateral-line hair cells viewed laterally, with close-up views of a single cell at the bundle region. The dashed green line indicates the single plane containing the stereocilia bundles. The magenta bracket indicates the area used to make the maximum projections that were analyzed for FM fluorescence in (F). (H) Sample traces of extracellular (microphonic) recordings, evoked from the inner ear of 3 dpf larvae. A piezo actuator was used to stimulate larvae with a 200 Hz sine-wave mechanical stimulus using an 8 V driver voltage. All statistics are mean ± SD, ****p < 0.0001. Scale bars are 10μm.

The literature on TMIE’s role in sensory hair cells is somewhat contradictory. Earlier studies proposed a developmental role for TMIE [21–23], while later studies evidenced a role in MET [26, 27]. To begin our analysis and attempt to clarify the issue in zebrafish, we examined live *tmie*^*ru1000*^ larvae at 5–7 days postfertilization (dpf) using confocal microscopy. The *ru1000* allele harbors a nonsense mutation leading to an N-terminal truncation, L25X [22]. We observed that mature hair cells of *tmie*^*ru1000*^ larvae were grossly normal compared to wild type siblings in both the inner ear lateral crista and the lateral line organ, an organ specific to fish and amphibians (Fig 1A). We detected a slight thinning of the mutant hair bundles, as revealed using a transgene *Actin-GFP* [34]. The underlying reason for the observed bundle thinning is not known, however we note that thin bundles have been observed in other zebrafish MET mutants such as those carrying mutations in *ap1b1* [35] and *tomt* [11]. Both genes have been previously implicated in protein trafficking in hair cells, with *tomt* having a specific role in targeting Tmc1/2 proteins to the hair bundle [11, 35]. We conclude that morphology is grossly normal in *tmie*-deficient zebrafish, suggesting that *tmie* does not have a developmental role in hair bundle formation. Our findings are consistent with the grossly normal hair-bundle morphology observed in *Tmie-/-* mice [26].

### Tmie-deficient zebrafish are deaf due to a defect in hair-cell mechanosensitivity

Next, we used an assay for the auditory evoked behavior response (AEBR) to quantify hearing loss in *tmie*^*ru1000*^ mutants. We exposed 6 dpf larvae to a pure tone stimulus (157 dB, 1000 Hz, 100 ms) once every 15 seconds for three minutes and recorded their startle responses (sample traces in Fig 1B). Larvae deficient in *tmie* appeared to be profoundly deaf, with little to no response compared to wild type siblings (Fig 1B and 1C). We then determined basal (unevoked) hair-cell activity of *tmie*^*ru1000*^ larvae using FM 1–43 or FM 4–64. Both are vital dyes that permeate open MET channels, making them useful for detecting the presence of active MET channels in hair cells [28, 29, 34]. A 30-second bath application of FM dye readily labels hair cells of the lateral line organ, which are arranged in superficial clusters called neuromasts. We briefly exposed wild type and *tmie*^*ru1000*^ larvae to FM dye and then imaged the neuromasts (Fig 1D). Consistent with previous findings [22, 23, 27], *tmie*^*ru1000*^ neuromasts have a severe reduction in FM labeling, suggesting that these hair cells have a MET defect. To characterize mechanically evoked responses of hair cells, we recorded extracellular potentials, or microphonics (Fig 1H). Using a piezo actuator, we applied a 200 Hz sine wave stimulus to 3 dpf larvae while simultaneously recording voltage responses from hair cells of the inner ear. In agreement with results from our FM dye assay and with microphonic recordings previously reported [22], microphonics are absent in *tmie*^*ru1000*^ larvae (Fig 1H, gray trace).

### Transgenic *tmie-GFP* rescues the functional defect in *tmie*^*ru1000*^ mutants

To rescue mechanosensitivity in *tmie*^*ru1000*^ larvae, we generated a construct expressing Tmie tagged with GFP on its C-terminus, then expressed this transgene using a hair cell-specific promoter, *myosin 6b (myo6b)*. Our Tmie-GFP rescued the FM labeling in *tmie*^*ru1000*^ hair cells (Fig 1E, quantified in Fig 1F). Tmie-GFP also restored microphonic potentials to wild-type levels (Fig 1H, orange trace). In a stable line with a single transgene insertion, we observed that Tmie-GFP expression varies among hair cells, even within the same patch of neuroepithelium (lateral crista, S1A Fig). Immature hair cells, which can be identified by their shorter stereocilia and kinocilia (S1A Fig, bracket and arrow, respectively), consistently show a bright and diffuse pattern of labeling. This high expression level in immature bundles is characteristic of transgenes expressed using the *myo6b* promoter, which drives expression more strongly in young hair cells [18, 34]. In mature hair cells, expression patterns of Tmie-GFP are variable. At high expression levels, Tmie-GFP signal is enriched in the bundle in a broader pattern (S1B Fig). At reduced levels, the signal appears to be concentrated at the beveled edge of the hair bundle (S1C Fig). At very low levels, we can observe puncta along the stereocilia staircase, consistent with localization at stereocilia tips (S1D Fig). We suspect that the diffuse “bundle fill” pattern is due to overexpression, and that lower levels of Tmie-GFP recapitulate the endogenous pattern of localization at the site of MET, as previously observed in mice [26].

### Tmie-GFP is capable of trafficking without other members of the MET complex

**Fig 2:**
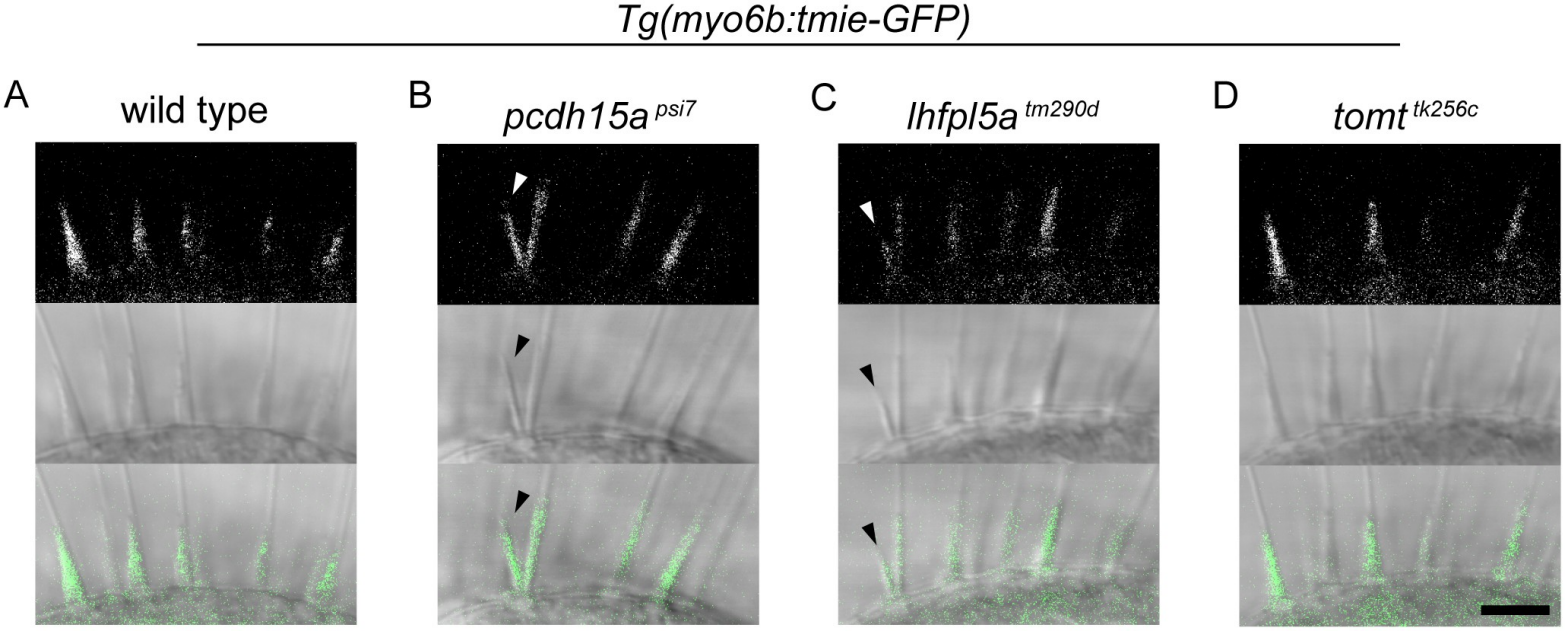
Tmie-GFP is present in the hair bundles of MET mutants. Confocal images of the bundle region in hair cells of the inner ear lateral cristae in 6 dpf larvae. Larvae expressing transgenic Tmie-GFP in the genetic backgrounds of wild type (A), and homozygous mutants for the tip link protein Pcdh15a (B, *pcdh15a*^*psi7*^), the accessory protein Lhfpl5a (C, *lhfpl5a*^*tm290d*^), and the Golgi-localized protein Tomt (D, *tomt*^*tk256c*^). Tomt-deficient fish lack Tmc expression in hair cell bundles [11], presumably mimicking the condition of a triple Tmc knockout. Arrowheads indicate splayed hair bundles. n = 8 each genotype. Scale bar is 5μm.

Having confirmed that our exogenously expressed Tmie-GFP is functional, we used this transgene to probe Tmie’s role in the MET complex. First, we characterized Tmie’s interactions with other MET proteins *in vivo* by expressing transgenic Tmie-GFP in mutant *pcdh15a*, *lhfpl5a*, and *tomt* larvae (Fig 2). Because a triple knock-out of the zebrafish *tmc* genes is not available, we used *tomt* mutants as a proxy for *tmc*-deficient fish based on recent reports of defective Tmc bundle localization in *tomt*-deficient fish and mice [11, 36]. As in wild type bundles (Fig 2A), we detected Tmie-GFP signal in each of these MET mutants (Fig 2B–2D), even in splayed hair bundles (Fig 2B and 2C, arrowheads). While normal localization of Tmie in the MET mutants could be in part due to overexpression, the presence of Tmie-GFP in stereocilia suggests that Tmie does not absolutely require any individual MET protein for entry into the hair bundle.

### Tmc1-GFP and Tmc2b-GFP fail to localize to stereocilia without Tmie

**Fig 3:**
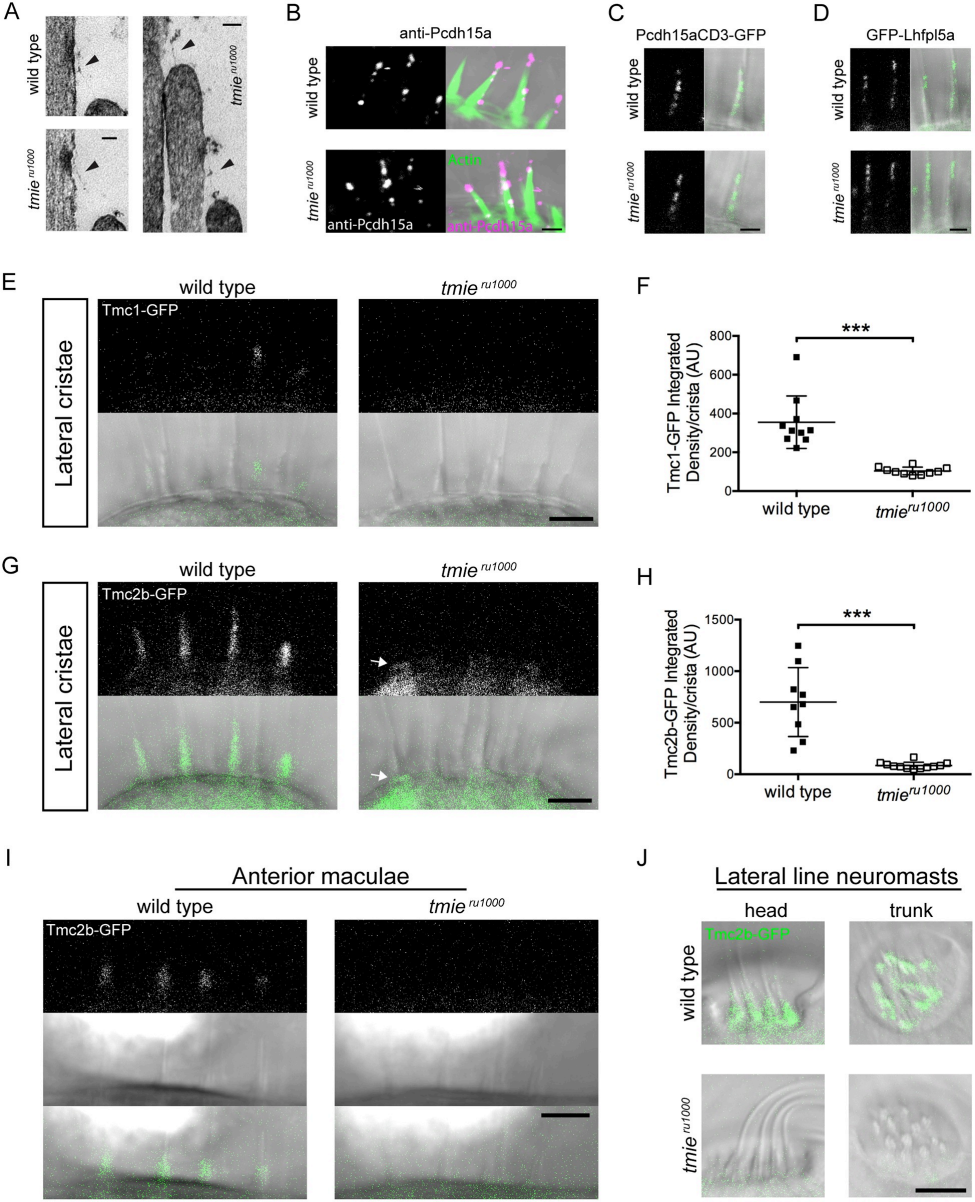
Specific loss of Tmc1 and Tmc2b in *tmie*^*ru1000*^ larvae. Images in A were collected using a transmission electron microscope (TEM). Images in B-J were collected using confocal microscopy. (A) TEM sections stained with 4% uranyl acetate and lead citrate. (B) Antibody labeling of Pcdh15a (magenta). Phalloidin was used to label actin (green). (C, D) Hair cells from the lateral cristae in 6 dpf larvae expressing either transgenic Pcdh15aCD3-GFP (C) or GFP-Lhfpl5a (D) (n = 6 each genotype). (E) Images of the lateral cristae in 4 dpf larvae expressing Tmc1-GFP. (F) Plot of the integrated density of Tmc1-GFP fluorescence in the ROI, expressed in arbitrary units; each data point represents one crista. Statistical significance determined by two-tailed unpaired t-test with Welch’s correction, p = 0.0002. (G) Images of the lateral cristae in 4 dpf larvae expressing Tmc2b-GFP. The arrow points to the cuticular plate/apical soma region, just below the ROI. (H) Plot of the integrated density of Tmc2b-GFP fluorescence in the ROI, expressed in arbitrary units. Statistical significance determined by two-tailed unpaired t-test with Welch’s correction, p = 0.0005. (I) Representatives images of anterior maculae in 2 dpf larvae expressing Tmc2b-GFP. We examined n = 14 wild type and n = 13 *tmie*^*ru1000*^ maculae. (J) Representative images of lateral line neuromasts in 4 dpf larvae expressing Tmc2b-GFP. We examined n = 18 wild type and n = 20 *tmie*^*ru1000*^ neuromasts. All statistics are mean ± SD. Scale bar in A is 50 nm, in B-D are 2 μm, in E-J are 5μm.

We next wanted to test whether loss of Tmie affects the integrity of the MET complex. To confirm the presence of tip links, we examined TEM images from 5 dpf wild type and *tmie*^*ru1000*^ larvae, n = 3 each (Fig 3A). Of 67 wild type sections examined, we observed 22 tip links, 23 insertion plaques, and 36 examples of tenting. Of 87 *tmie*^*ru1000*^ sections examined, we observed 27 tip links, 26 insertion plaques, and 39 examples of tenting. We then used an antibody against the tip-link protein Pcdh15a and observed punctate expression along stereocilia in *tmie*^*ru1000*^ larvae (Fig 3B). Finally, we stably expressed GFP-tagged Pcdh15aCD3 and its trafficking partner, Lhfpl5a, in *tmie*^*ru1000*^ larvae. Stable expression of transgenes in zebrafish is achieved through random genomic insertion of a plasmid containing promoter and gene. To ascertain comparable expression of a given transgene across larvae, we used transgenic lines with 50% transmission, indicative of a single insertion event. Siblings produced in the same clutch of eggs were used as controls throughout this study. In 6 dpf *tmie*^*ru1000*^ larvae, we observed punctate expression of transgenic Pcdh15aCD3-GFP (Fig 3C) and GFP-Lhfpl5a (Fig 3D) along stereocilia tips, similar to the pattern obtained with antibody labeling of Pcdh15a. These three assays confirmed that tip links were intact in *tmie*^*ru1000*^ larvae, which agrees with our *in vivo* data (Fig 1A) and previous results in *Tmie-/-* mice [26].

To test for the presence of the Tmc proteins in *tmie*^*ru1000*^ stereocilia, we again used stable GFP-tagged transgenic lines with single insertions: *Tg(myo6b*:*tmc1-GFP)* and *Tg(myo6b*:*tmc2b-GFP)* [11]. Unfortunately, we were unable to successfully express a *tmc2a* transgene. The Tmc1-GFP signal was very dim and only reliably visualized in a subset of the tall and accessible hair bundles of the lateral cristae, where we detected severely reduced GFP fluorescence in the stereocilia of *tmie*^*ru1000*^ hair cells as compared to wild type siblings (Fig 3E). The Tmc2b-GFP signal was more robust and we detected it in the hair bundles of the lateral cristae (Fig 3G), anterior maculae (Fig 3I), and lateral line organ (Fig 3J). We imaged anterior maculae at 2 dpf, which are closer the surface of the fish at this stage of development; the GFP signal was too faint in the posterior maculae, which are located in a deeper, medial position next to the brain. In all of these hair cell types, we observed a severe reduction in Tmc2b-GFP fluorescence in the hair bundles of *tmie*^*ru1000*^ larvae. Although Tmc2b was previously reported to have differential effects in lateral line neuromasts [9], we did not observe a difference in Tmc2b-GFP expression among head or trunk neuromasts, likely because the *myo6b* promoter drives expression in all hair cells. In the lateral cristae, mature *tmie*^*ru1000*^ hair cells expressing Tmc2b-GFP often displayed fluorescence within the apical soma near the cuticular plate, suggesting a trafficking defect (Fig 3G, arrows; position of cuticular plate denoted in Fig 1G). We quantified the loss of Tmc1-GFP (Fig 3F) and Tmc2b-GFP (Fig 3H) from the hair bundle region of lateral cristae and found a striking and consistent reduction in *tmie* mutants. Loss of Tmc1/2b could be a result of disruption of the MET complex, but we previously showed that localization of transgenic Tmc1-GFP and Tmc2b-GFP is normal in *pcdh15a* mutants [11]. The aforementioned study and our experiments demonstrated that mislocalization of Tmc1/2b is not a hallmark of all MET mutants, and thus their mislocalization in *tmie* mutants is a specific effect.

### Overexpression of Tmie increases bundle localization of Tmc1-GFP and Tmc2b-GFP

**Fig 4:**
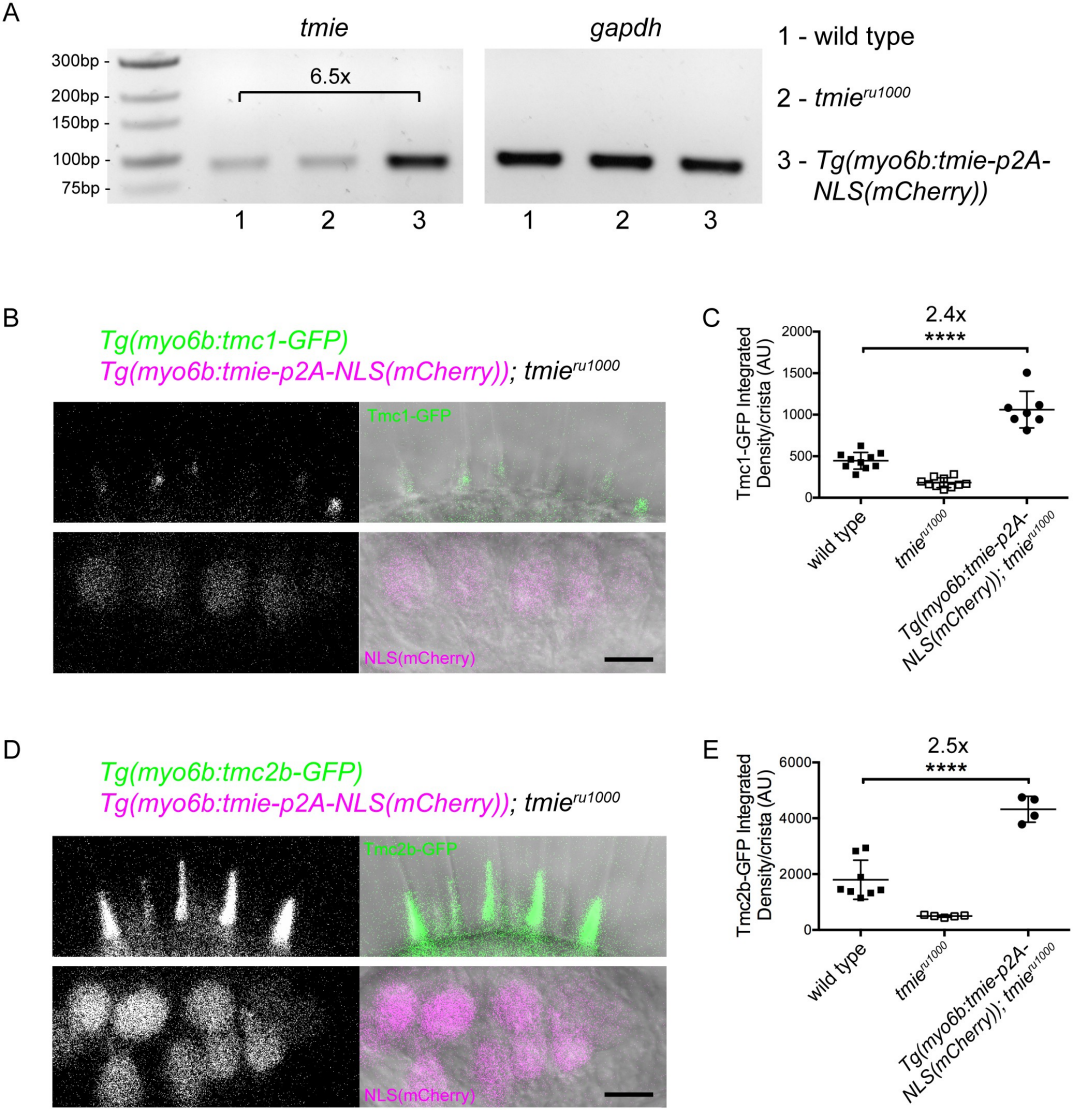
Overexpression of Tmie causes increased bundle expression of Tmc1-GFP and Tmc2b-GFP. (A) Semi-quantitative PCR of *tmie* cDNA using primers within exon 4 to detect transcripts from both transgene and the endogenous gene. We generated cDNA from RNA extracted from 5 dpf whole larvae. Larvae carrying *Tg(myo6b*:*tmie-p2A-NLS(mCherry))* had 6.5-fold more *tmie* transcript than wild type non-transgenic siblings. Products are from 40 cycles. Less PCR reaction (2.5x less) was loaded for *gapdh* to avoid saturation. (B) Confocal images of the lateral cristae in 4 dpf *tmie*^*ru1000*^ larvae co-expressing two transgenes, *tmc1-GFP* (upper panel) and *tmie-p2A-NLS(mCherry)* (lower panel). The p2A linker is a self-cleaving peptide that results in equimolar translation of Tmie and nuclear mCherry. (C) Plot of the integrated density of Tmc1-GFP fluorescence/crista in the ROI, expressed in arbitrary units; significance determined by one-way ANOVA. (D-E) Same as B-C except using *tmc2b-GFP* instead of *tmc1-GFP*. All statistics are mean ± SD. Scale bars are 5μm.

We hypothesized that if the loss of Tmie reduces Tmc localization in the hair bundle, then overexpression of Tmie would have the opposite effect. To test the consequence of overexpression of Tmie on Tmc localization, we created a second construct of *tmie* coupled with *p2A-NLS(mCherry)*. The p2A linker is a self-cleaving peptide, which leads to translation of equimolar amounts of Tmie and NLS(mCherry). Hence, mCherry expression in the nucleus denotes Tmie expression in the cell (Fig 4B and 4D, lower panels). We generated a stable *tmie*^*ru1000*^ fish line carrying the *tmie-p2A-NLS(mCherry)* transgene driven by the *myo6b* promoter. Semi-quantitative PCR revealed that the *myo6b* promoter produces higher transcript levels of *tmie* than in non-transgenic siblings (Fig 4A). We then crossed *tmie*^*ru1000*^ fish carrying *Tg(myo6b*:*tmie-p2A-NLS(mCherry))* to *tmie*^*ru1000*^ fish carrying either *Tg(myo6b*:*tmc1-GFP)* or *Tg(myo6b*:*tmc2b-GFP)*. In the lateral cristae, we observed that overexpression of Tmie led to a robust increase in bundle expression of both Tmc1-GFP (Fig 4B) and Tmc2b-GFP (Fig 4D). We quantified GFP fluorescence in the hair bundle region of *tmie*^*ru1000*^ larvae and found that, compared to wild type siblings expressing only one of the *tmc-GFP* transgenes, co-overexpression with Tmie increased bundle expression of Tmc1-GFP by 2.4-fold (Fig 4C) and Tmc2b-GFP by 2.5-fold (Fig 4E). Combined with the finding that Tmc expression is lost in hair bundles lacking Tmie, our data suggest that Tmie positively regulates Tmc localization to the hair bundle.

We questioned whether Tmie was affecting the level of translation of *tmc1/2b* transcripts, but examination of GFP fluorescence in the soma region of the lateral crista revealed no difference between *tmie*^*ru1000*^ and sibling larvae expressing the Tmc2b-GFP transgene (S2A Fig). There was also no difference in whole-cell GFP fluorescence (S2B Fig). Subtracting the soma signal from the whole cell signal revealed that the difference was in the bundle region (S2C Fig). This difference is likely not detected in whole cell fluorescence because the relative contribution of signal from the bundle is small. Our observations were similar when we examined soma fluorescence from *tmie*^*ru1000*^ larvae co-expressing Tmie and Tmc2b-GFP (S2D–S2F Fig). These results indicate that Tmie is unlikely to affect translation of *tmc* transcripts, reinforcing our hypothesis that Tmie regulates Tmc bundle localization.

### Transgenes can effectively determine protein functional capacity

To gain a better understanding of Tmie’s role in regulating the Tmcs, we characterized a new allele of *tmie*, *t26171*, which was isolated in a forward genetics screen for balance and hearing defects in zebrafish larvae. Sequencing revealed that *tmie*^*t26171*^ fish carry an A→G mutation in the splice acceptor of the final exon of *tmie*, which leads to use of a nearby cryptic splice acceptor (S3A Fig, *DNA*, *cDNA*). Use of the cryptic acceptor causes a frameshift that terminates the protein after amino acid 139 (A140X), thus removing a significant portion of the cytoplasmic C-terminus (S3A Fig, *Protein*). Homozygous mutant larvae exhibit severe auditory and vestibular deficits, being insensitive to acoustic stimuli and unable to maintain balance (S3A Fig, *Balance*). FM 4–64 labeling of *tmie*^*t26171*^ mutant hair cells suggests that the effect of the mutation is similar to the *ru1000* mutation (S3B Fig, quantified in S3D Fig). This finding implicates the C-terminal tail, a previously uncharacterized region, in Tmie’s role in MET. However, when we overexpressed a near-mimic of the predicted protein product of *tmie*^*t26171*^ (1-138-GFP) using the *myo6b* promoter, we observed full rescue of FM labeling defects in *tmie*^*ru1000*^ larvae (S3C Fig, quantified in S3D Fig), as well as behavioral rescue of balance and acoustic sensitivity (n = 19). These results revealed that when expressed at higher levels, loss of residues 139–231 does not have a significant impact on Tmie’s ability to function.

**Fig 5:**
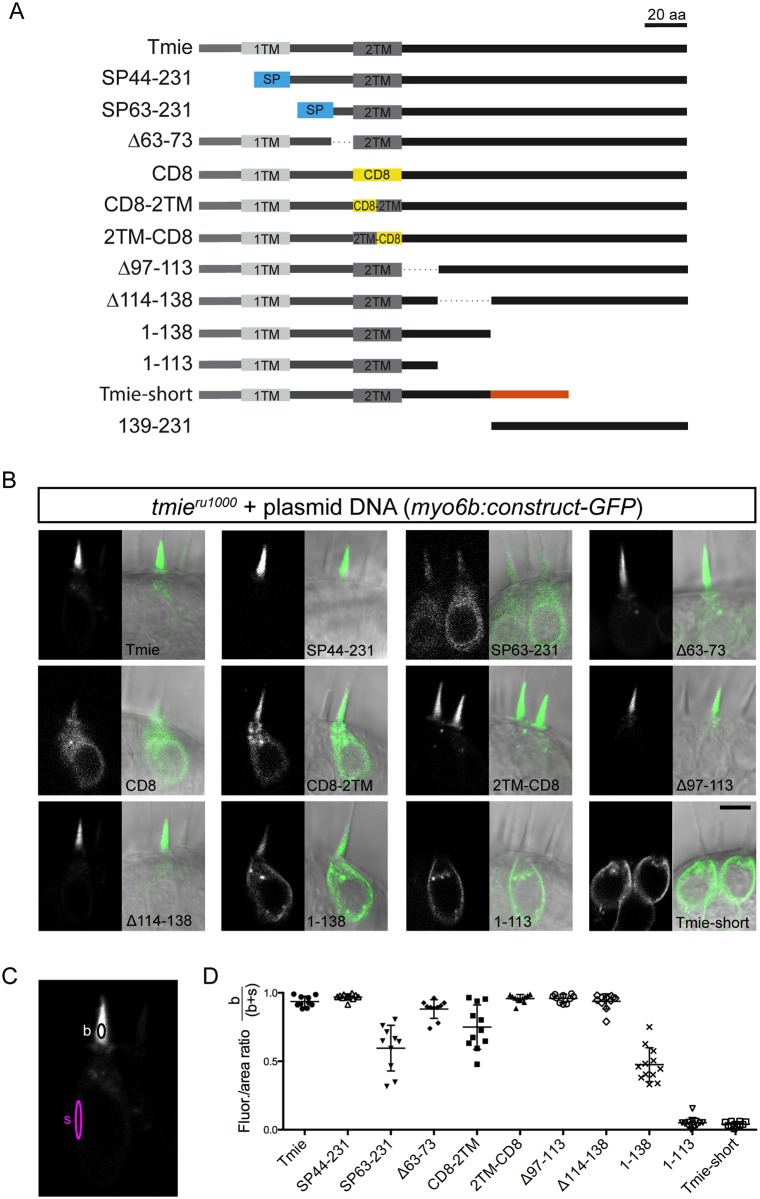
Schema for a systematic domain analysis of Tmie and subcellular localization of Tmie constructs. (A) A linear diagram of 13 unique constructs of Tmie used in our experiments. Full-length zebrafish Tmie contains two hydrophobic regions predicted to form transmembrane helices (1TM and 2TM). SP44-231 and SP63-231 replace part of the N-terminus with a signal peptide (SP) from the Glutamate receptor 2a (in blue). In the CD8, CD8-2TM, and 2TM-CD8 constructs, all or part of the 2TM is replaced by the helix from the CD8 glycoprotein (in yellow). Tmie-short is a fish-specific isoform of Tmie that contains an alternate final exon (in orange). Dotted lines represent internal deletions. (B) Representative confocal images of each construct being expressed as a GFP-tagged transgene in hair cells of 4–6 dpf *tmie*^*ru1000*^ larvae. Expression is mosaic due to random genomic insertion into subsets of progenitor cells after single-cell injection. All images are of cells in the inner ear cristae. Scale bar is 5μm. (C) The localization of each GFP fusion protein was determined by measuring the fluorescence/area in the bundle (b) and soma (s), and then calculating b / (b+s). (D) Enrichment in the hair bundle is displayed as a ratio for each construct, with 1 being completely bundle-enriched and 0 being completely soma-enriched.

This paradoxical finding highlighted an important advantage of the use of transgenes over traditional mutants when identifying domains that are fundamentally essential to the function of a protein. There are myriad reasons why a genomic mutation may lead to dysfunction, including reduced transcription or translation, protein misfolding and degradation, or mistrafficking. In cases where a mutated protein retains partial efficacy, exogenous expression may overcome these deficiencies by producing proteins at higher levels. This overexpression can reveal domains that are truly essential or non-essential to protein function, as seen with the differential rescue results in the *tmie*^*t26171*^ mutant and its transgene mimic (S3D Fig). Moreover, the use of transgenes enabled us to carry out a comprehensive structure/function analysis of Tmie. To this end, we systematically deleted or replaced regions of *tmie* to generate 13 unique *tmie* constructs (Fig 5A), and then expressed these constructs in hair cells of the *tmie*^*ru1000*^ mutants.

Earlier studies in zebrafish and mice proposed that Tmie undergoes cleavage, resulting in a single-pass mature protein [22, 37]. To test this hypothesis, we generated the SP44-231 construct of Tmie, which replaced the N-terminus with a known signal peptide (SP) from a zebrafish Glutamate receptor protein (Gria2a). The unrelated signal peptide serves to preserve the predicted membrane topology of Tmie. We also generated a similar construct that begins at amino acid 63, where the sequence of Tmie becomes highly conserved (*SP63-231*). Three of the constructs contained internal deletions (*Δ63–73; Δ97–113; Δ114–138)*. In three more constructs, we replaced part of or the entire second transmembrane helix (2TM) with a dissimilar helix from the CD8 glycoprotein (*CD8; CD8-2TM; 2TM-CD8*). We included our mimic of the zebrafish *tmie*^*t26171*^ mutant, which truncates the cytoplasmic C-terminus (*1–138*). Further manipulating the C-terminus, we made a construct that mimics the truncation seen in the mouse *sr*^*J*^ mutant (*1–113*). In mice, this truncation recapitulates the phenotype seen in a complete deletion of *Tmie* [21]. In addition, the *Δ114–138* construct deletes the internal region of *tmie* that differentiates constructs *1–113* and *1–138*. We included an alternate splice isoform of *tmie* with a different final exon, altering the C-terminal sequence (*Tmie-short*). This isoform is found only in zebrafish [22] and its function has not been explored. Finally, we expressed a fragment of the C-terminus that is lost in our zebrafish *tmie*^*t26171*^ mutant (*139–231*).

### Subcellular localization of mutated or chimeric Tmie reveals domains required for self-localization to the bundle

To determine subcellular localization of the transgenic *tmie* constructs, we inserted the coding sequence of each construct into a plasmid containing the *myo6b* promoter for expression, including a C-terminal GFP tag for visualization. These plasmids were then individually co-injected into *tmie*^*ru1000*^ eggs with transposase mRNA to generate mosaic expression of the constructs in a subset of hair cells. At 4–6 days post injection, we imaged hair cells expressing each transgene (Fig 5B). To quantify the enrichment in the bundle versus soma, we measured the integrated density of GFP fluorescence in a small central area of mature bundles (Fig 5C, black oval) and separately in the plasma membrane or soma-enriched compartments (Fig 5C, magenta oval). Correcting for area, we then divided the bundle values by the total values (bundle/bundle + soma) and expressed this as a ratio (Fig 5D). Values closer to 1 are bundle enriched, while values closer to 0 are soma-enriched. We excluded two constructs from this analysis: CD8-GFP because it was detected only in immature bundles (Fig 5B, *CD8*, and S4A Fig), and 139-231-GFP because it filled all regions of the hair cell (S4C Fig).

Localization fell into three broad categories: bundle-enriched, soma-enriched, and equally distributed. Most of the fusion proteins were bundle-enriched, similar to full-length Tmie-GFP expression (Fig 5B and 5D). Three constructs were trafficked to the bundle but also expressed strongly in the soma (*SP63-231*, *CD8-2TM*, *1–138*). This result suggests that the deleted regions in these constructs have some role in designating Tmie as a bundle-localized protein. Also of note, the full replacement of the 2TM helix (*CD8*) was unable to maintain stable expression in mature bundles (S4A Fig), and did not show rescue of FM label in lateral line hair cells of *tmie*^*ru1000*^ larvae (S4B Fig). Half-TM replacements (*CD8-2TM*, *2TM-CD8*) revealed that loss of the first half of the helix affects trafficking, whereas alteration of the second half had no effect. Only two constructs that included the second TM were soma-enriched (*Tmie-short* and *1–113*), suggesting an inability to traffic to the bundle. These two constructs were thus excluded from further analyses. Since three of our constructs that manipulated the C-terminus showed impaired bundle targeting, we expressed a fragment of the C-terminus (*139-231-GFP*) to determine if it contained a bundle targeting signal. Expression of this fragment was restricted to the soma and kinocilium with little to no bundle expression (S4C Fig), and showed no functional rescue of MET activity (S4D Fig). Together, our results suggest that no single motif but rather multiple regions of Tmie contribute to its bundle localization.

### FM labeling identifies functional regions in the second transmembrane domain and adjacent residues of Tmie

**Fig 6:**
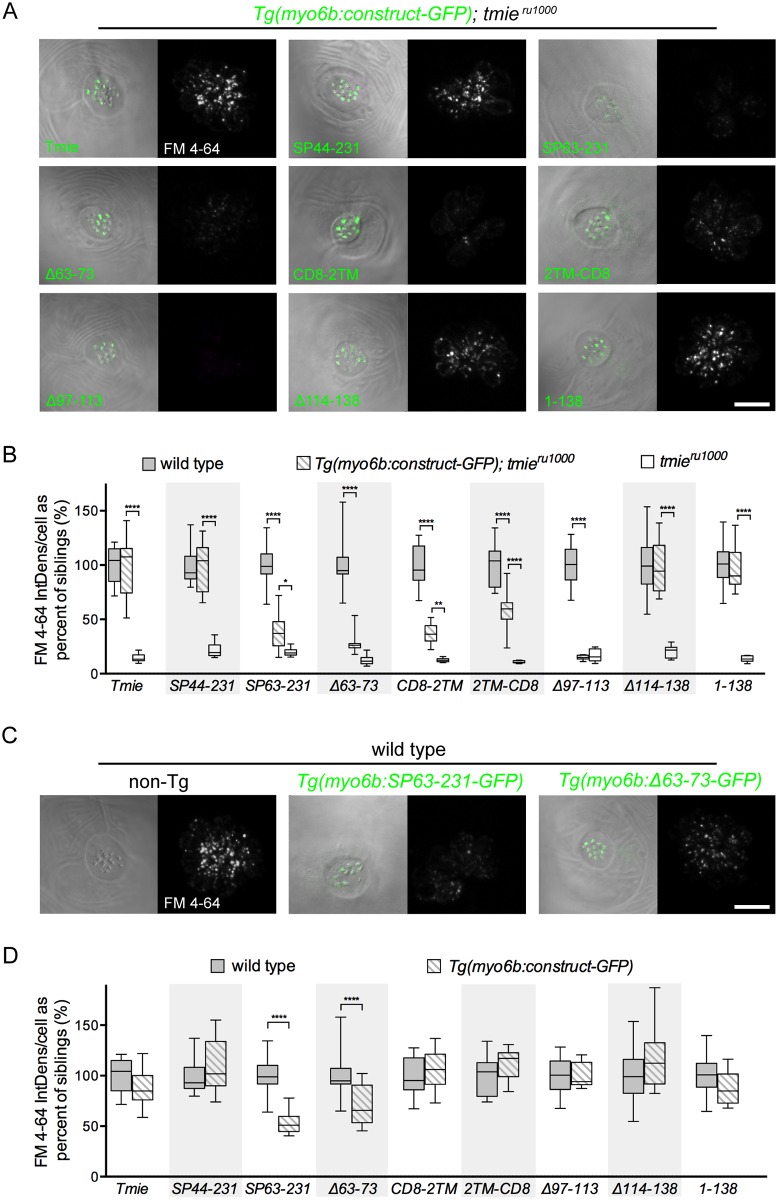
The second transmembrane and adjacent residues of Tmie are required for rescue of FM labeling. All images are a top-down view of a representative neuromast from 6 dpf larvae collected using confocal microscopy. The left image is a single plane through the stereocilia (green dashed line in Fig 1G) with DIC + GFP fluorescence. The right image is a maximum projection of the 7 sections in the soma region (magenta bracket in Fig 1G) showing FM 4–64 fluorescence. (A) Representative images of neuromasts in *tmie*^*ru1000*^ larvae, each stably expressing an individual *tmie* construct. FM fluorescence was normalized to wild type non-transgenic larvae generated with the Tmie-GFP line. (B) Box-and-whiskers plot of the integrated density of FM fluorescence/cell for each *tmie* construct. We normalized values to the average of wild type siblings for each construct. (C) Representative images of neuromasts in wild type larvae with or without transgene. FM fluorescence was normalized to wild type non-transgenic larvae of the Tmie-GFP line. (D) Box-and-whiskers plot of the integrated density of FM fluorescence/cell in wild type neuromasts with and without *tmie* transgene. We normalized values to the average of wild type siblings for each construct. Significance determined within each clutch by one-way ANOVA, n ≥ 9, **p < 0.01, ***p < 0.001, ****p < 0.0001. Scale bars are 10μm.

To identify regions of Tmie involved in the mechanosensitivity of hair cells, we measured the functionality of the nine *tmie* constructs that yielded hair-bundle expression. As in Fig 1F, we generated stable lines of each transgenic construct and quantified fluorescence in lateral line neuromasts after exposure to FM 4–64 (Fig 6). We used larvae at 6 dpf, a later stage that maximizes the number of hair cells in each neuromast. In all but one case (*CD8-2TM-GFP*), these fish lines contained single transgene insertions to equalize expression of the *tmie* construct within a clutch. In the case of *CD8-2TM-GFP*, we used larvae from a founder that transmitted the transgene to >10% of offspring with consistently bright expression.

Of nine constructs examined, four generated wild type levels of FM fluorescence in *tmie*^*ru1000*^ neuromasts (*Tmie*, *SP44-231*, *Δ114–138*, and *1–138*; Fig 6A and 6B). Two constructs (*Δ97–113* and *Δ63–73*) did not rescue above mutant levels of FM 4–64. While residues 63–73 have not been characterized, the *Δ97–113* result is consistent with the findings of previous publications in humans and mice, showing that mutations in this intracellular region impair hearing and hair cell function [24, 26]. Three constructs were capable of producing partial rescue (*SP63-231*, *CD8-2TM*, and *2TM-CD8*). Each one of the five dysfunctional constructs altered part of a contiguous region of Tmie: the 2TM and adjacent domains. These results highlight this region of Tmie as vital for function. To determine whether any of the constructs also produce a dominant effect on hair-cell function, we compared FM label in wild type larvae with or without the individual transgenic *tmie* constructs (Fig 6D). Expression of GFP-tagged SP63-231 or Δ63–73, which yielded impaired rescue in *tmie*^*ru1000*^ larvae, caused reduced FM label in transgenic wild type cells (Fig 6C and 6D). Interestingly, both dominant negative constructs delete parts of the extracellular region of Tmie.

### Recordings of mechanically evoked responses confirm that the second transmembrane domain and adjacent regions are required for normal hair-cell function

**Fig 7:**
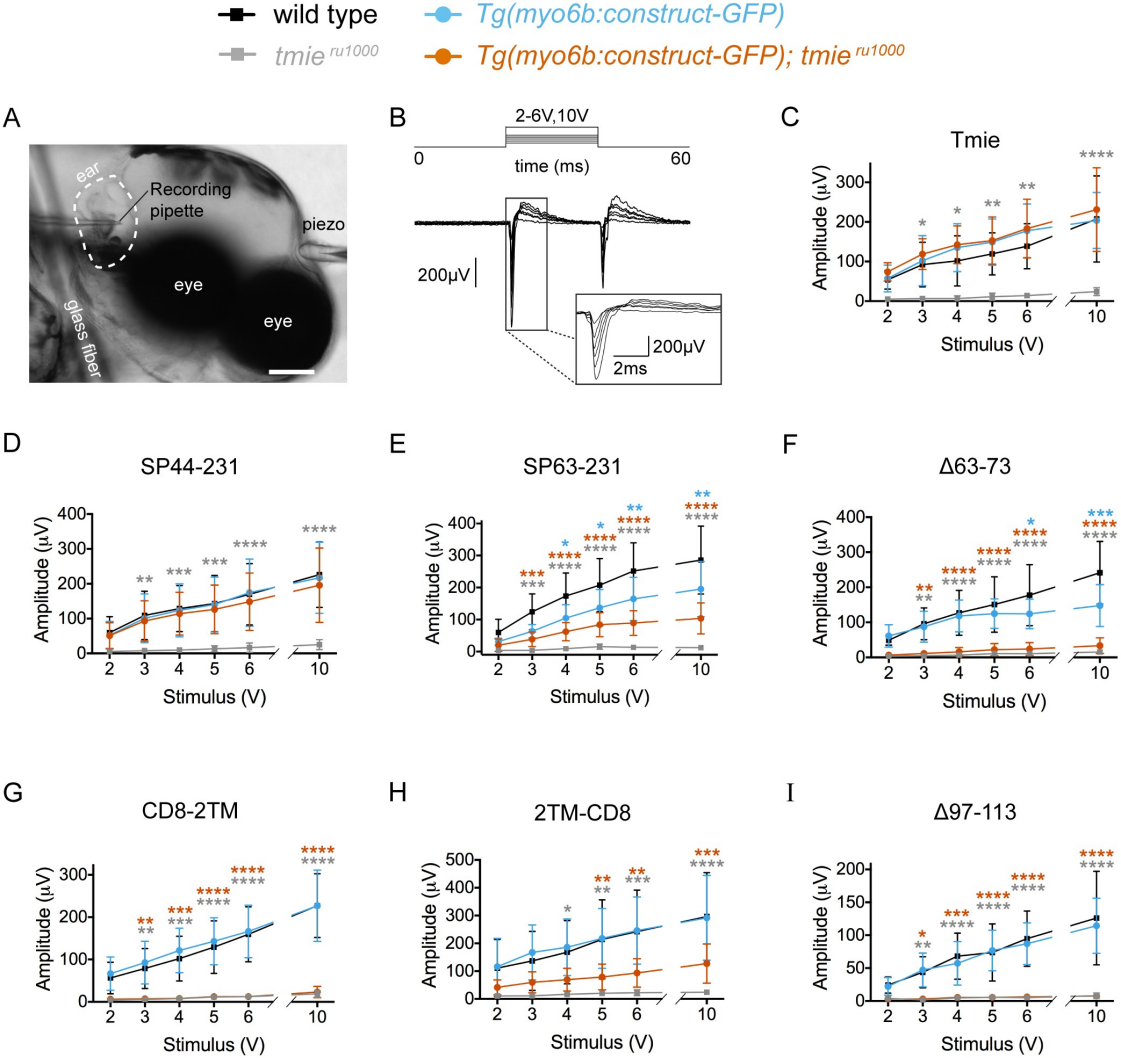
The second transmembrane and adjacent regions of Tmie are required for inner ear microphonics. (A) A DIC image of a 3 dpf larva, anesthetized and pinned (glass fiber) for inner ear recordings. Shown are a probe attached to a piezo actuator (piezo) pressed against the head and a recording pipette pierced into the inner ear. Scale bar is 100μm. (B) Example traces from a wild type larva. A step stimulus was applied with a 20 ms duration; 200 traces were averaged for each of the six piezo driver voltages: 2V, 3V, 4V, 5V, 6V, and 10V. Gray box: magnification of the onset of response in individual traces. (C-I) Plots of the mean amplitude of the response peak ± SD as a function of the stimulus intensity of the driver voltage. We used the stimulation protocol explained in B to obtain responses from larvae expressing one of our transgenic *tmie* constructs, as labeled. Statistical significance was determined by two-way ANOVA comparing all groups to wild type non-transgenic siblings, n ≥ 5, *p < 0.05, **p < 0.01, ***p < 0.001, ****p < 0.0001.

Bath applied FM dye demonstrates the presence of permeable MET channels, but does not reveal any changes in mechanically evoked responses in hair cells. Therefore, we also recorded microphonics of mutant larvae expressing individual *tmie* transgenes. Reduced hair-cell counts have been observed in neuromasts of MET mutants at 6 dpf but not at 2 dpf [11], however, the amplitude of microphonics increases with age and cell counts [38]. As a compromise, we used 3 dpf larvae and recorded from the inner ear where there is a larger population of hair cells. This earlier time point additionally allowed us to determine MET activity near the onset of mechanotransduction to rule out indirect or progressive effects of Tmie loss. We inserted a recording pipette into the inner ear cavity and pressed a glass probe against the head (Fig 7A). Using a piezo actuator to drive the probe, we delivered a step stimulus at increasing driver voltages while recording traces in current clamp (Fig 7B). For each transgenic *tmie* line, we measured the amplitude of the response at the onset of stimulus (Fig 7C–7I). We limited our analysis to the lines expressing constructs that failed to fully rescue FM labeling (Fig 7E–7I). As positive controls, we used the full-length *tmie-GFP* line (Fig 7C) and also included the *SP44-231-GFP* line (Fig 7D), expressing the cleavage product mimic. Both control constructs fully rescued the responses in *tmie*^*ru1000*^ larvae. Consistent with a reduction in labeling with FM dye, we found that the microphonic responses were strongly or severely reduced in *tmie*^*ru1000*^ larvae expressing the GFP-tagged constructs *SP63-231*, *Δ63–73*, *CD8-2TM*, *2TM-CD8* or *Δ97–113* (Fig 7E–7I). We again saw dominant negative effects in wild type larvae expressing transgenic *SP63-231-GFP* or *Δ63-73-GFP* (Fig 7E and 7F, blue traces).

### Regions of Tmie that mediate hair-cell mechanosensitivity are also required for localizing Tmc2b-GFP

**Fig 8:**
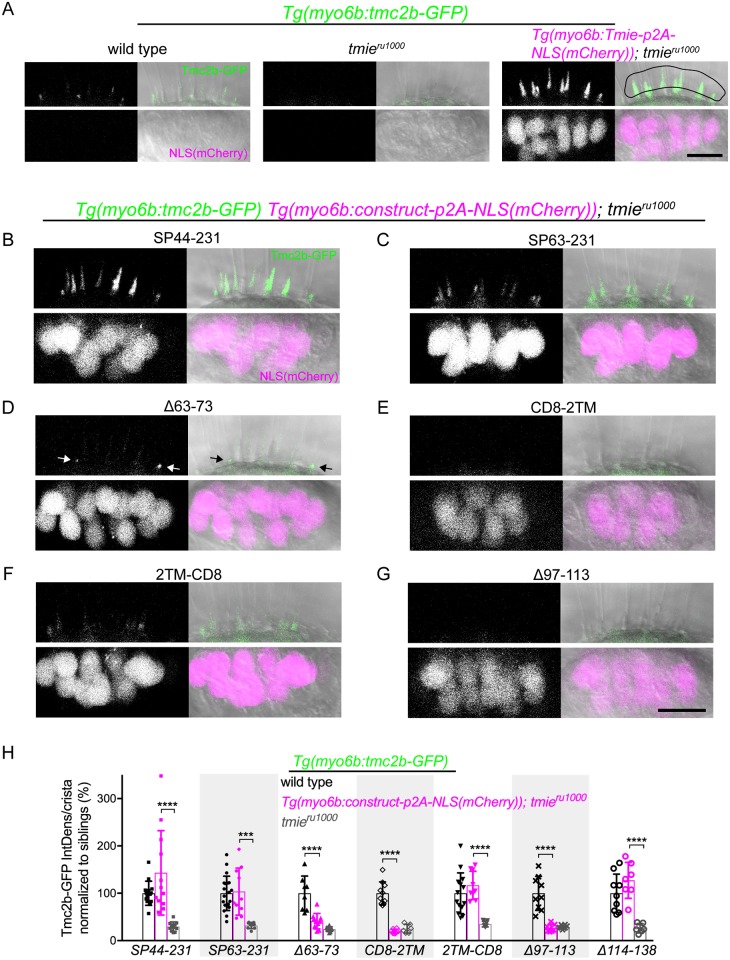
Effect of transgenic Tmie constructs on Tmc2b-GFP bundle localization. Confocal images are maximum projections of representative inner-ear lateral cristae collected from 4 dpf larvae. Upper panels show the bundle region, with all larvae stably expressing transgenic Tmc2b-GFP (green). Lower panels show the soma region, with some larvae expressing transgenic Tmie constructs tagged with p2A-NLS(mCherry). Nuclear mCherry (magenta) is a marker for equimolar translation of the indicated Tmie construct. Two *tmie* construct lines contained stable transgene insertions (SP44-231, CD8-2TM), whereas F1 larvae were used for the Δ63–73, Δ97–113, and Δ114–138 constructs; for the SP63-231 construct, we used a mix of larvae with stable insertions or F1 offspring. (A) Sibling wild type, *tmie*^*ru1000*^, and *tmie*^*ru1000*^ expressing transgenic Tmie. For the quantification in H, Tmc2b-GFP fluorescence was measured within the ROI (right panel, black line). (B-G) Images of lateral cristae from *tmie*^*ru1000*^ larvae expressing individual *tmie* constructs tagged with *p2A-NLS(mCherry)*, as labeled. The arrows in D point to Tmc2b-GFP in immature hair bundles. (H) Plot of the integrated density of Tmc2b-GFP fluorescence in the ROI, comparing *tmie*^*ru1000*^ larvae expressing a *tmie* construct (magenta) to wild type (black) and *tmie*^*ru1000*^ (gray) siblings not expressing *tmie* construct. We normalized values to the average of wild type siblings for each construct. Significance for SP44-231, SP63-231, and 2TM-CD8 was determined by the Kruskal-Wallis test, for all other *tmie* constructs by one-way ANOVA, n ≥ 6, ***p < 0.001, ****p < 0.0001. Scale bars are 10μm.

After identifying functional regions of Tmie, we asked whether these regions are involved in regulating Tmc localization. To answer this question, we quantified hair bundle expression of transgenic Tmc2b-GFP in hair cells of *tmie*^*ru1000*^ mutant larvae stably co-expressing individual transgenic *tmie* constructs (Fig 8H). As in Fig 4, we tagged our *tmie* constructs with *p2A-NLS(mCherry)* so that Tmc2b-GFP expression in the hair bundles could be imaged separately. Because we did not generate stable transgenic lines for all constructs, we were concerned that variable levels of Tmie protein expression among siblings, particularly low levels, might confound the experiment. However, examination of our lowest expressing *p2A-NLS(mCherry)* constructs (*Tmie*, *CD8-2TM*, and *Δ97–113*) revealed no correlation between the mCherry and GFP signals; higher levels of nuclear mCherry signal did not correlate with higher bundle expression of GFP-tagged Tmc protein (S5A Fig). Additionally, the *Tmie-p2A-NLS(mCherry)* line used in S5A Fig was also used for semi-qPCR as well as quantification of Tmc1-GFP signal. This low level of mCherry signal (visualized in Fig 4B, lower panel) corresponded to a 6.5-fold increase in *tmie* transcript (Fig 4A) and resulted in a 2.4-fold increase in Tmc1-GFP (Fig 4C), comparable to the 2.5-fold increase of Tmc2b-GFP (Fig 4E) seen in a different *Tmie-p2A-NLS(mCherry)* line with brighter mCherry signal (visualized in Fig 4D, lower panel). Taken together, these results indicate that even very low-expressing transgenes produce saturating levels of Tmie protein. We confirmed the lack of correlation between mCherry and Tmc2b-GFP signals with our other *tmie* constructs (S5B Fig). We then proceeded to examine bundle expression of Tmc2b-GFP when co-expressed with the positive control of *SP44-231* and the five *tmie* constructs that yielded impaired mechanosensitivity. As an additional positive control, we also included analysis of the deletion *Δ114–138*, a construct with full functional rescue and normal localization. As in Figs 3 and 4, we used the taller and more accessible bundles of the lateral crista for quantification.

Four constructs showed full rescue of Tmc2b-GFP levels in the bundles of *tmie*^*ru1000*^ larvae. The SP44-231 cleavage mimic produced highly variable levels with examples in the wild type range and others increasing Tmc2b-GFP expression well above wild type levels (Fig 8B and 8H). The higher levels of Tmc2b-GFP achieved with SP44-231 are comparable to the signal increase caused by overexpression of full-length Tmie (Figs 4D, 4E and 8A, right panel). We suspect that the exogenous Gria2a signal peptide leads to variable processing of SP44-231 and thus contributes to this variability in Tmc2b-GFP fluorescence. In *tmie*^*ru1000*^ larvae expressing the positive control construct *Δ114–138*, we observed values of Tmc2b-GFP fluorescence in the wild type range, as expected (Fig 8H, *Δ114–138*). Surprisingly, constructs *SP63-231* (Fig 8C) and *2TM-CD8* (Fig 8F) also gave rise to wild type levels of Tmc2b-GFP (Fig 8H). When we recorded microphonics in these larvae, we found that co-expression of Tmc2b-GFP with either SP63-231 (S6A Fig) or 2TM-CD8 (S6C Fig) resulted in better functional rescue of *tmie*^*ru1000*^ than when either *tmie* construct was expressed without Tmc2b-GFP (Fig 7E and 7H). We also determined that microphonic potentials correlated with the levels of Tmc2b-GFP in the bundles of *tmie*^*ru1000*^ larvae co-expressing 2TM-CD8, r = 0.879, p = 0.0018 (S6D Fig). The same analysis of SP63-231 showed a positive trend, r = 0.722, yet was statistically non-significant (p = 0.1682), which may be due to small sample size (S6B Fig). These results suggest that in the lines of SP63-231 and 2TM-CD8, functional rescue is Tmc dose-dependent.

Of the three constructs that produced little to no functional rescue, CD8-2TM (Fig 8E) and Δ97–113 (Fig 8G) had severely reduced levels of Tmc2b-GFP in hair bundles (Fig 8H). In *tmie*^*ru1000*^ larvae expressing the *Δ63–73* construct, there was severely reduced but still faintly detectable Tmc2b-GFP signal (Fig 8D). As with the functional rescue experiments, this difference was not statistically significant (Fig 8H). Interestingly, the bulk of this signal was observed in immature bundles (Fig 8D, arrows, and S7 Fig), but there was some detectable Tmc2b-GFP signal in mature bundles (S7 Fig). To generalize our findings to Tmc1, we examined Tmc1-GFP localization in *tmie*^*ru1000*^ larvae co-expressing the null-function construct CD8-2TM (S8 Fig). As with Tmc2b-GFP, we detected no bundle expression of Tmc1-GFP. Overall, these results suggest that the level of functional rescue by the *tmie* constructs is correlated to the amount of Tmc1/2b present in the hair bundle.

## Discussion

**Fig 9:**
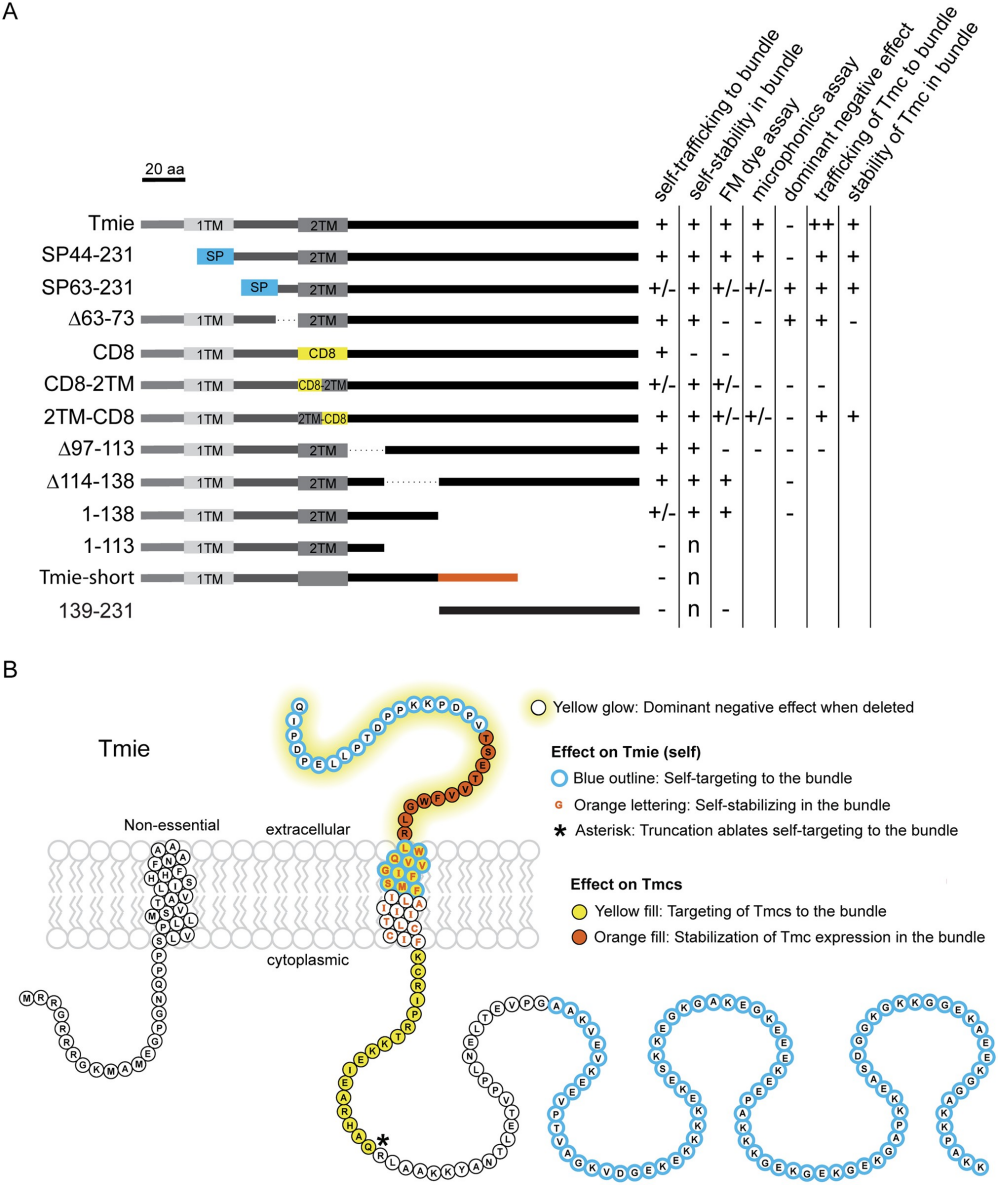
Summary of experimental results for *tmie* constructs and model of discrete functional domains of Tmie. (A) Symbols as follows: enhanced above wild type (++), comparable to wild type (+), partially reduced (+/-), severely reduced or absent (-), and not applicable (n). For *dominant negative effect*, the effect is present (+) or absent (-). Blank spaces were determined for reasons discussed in the text. Refer to Fig 5A for details on constructs. (B) A model of the protein sequence of zebrafish Tmie. Amino acids 1–43 are separated due to suspected cleavage as a signal peptide. Although shown as a TM domain, it is unclear whether the first hydrophobic region forms a helix. Note that the extracellular region (yellow glow) was never deleted in its entirety; SP63-231 deleted QIPDPELLPTDPPKKPDPV, and Δ63–73 deleted TSETVVFWGLR. Also note that the TM domain (orange lettering) only had an effect on the stability of Tmie when the entire helix was substituted.

*TMIE* was first identified as a deafness gene in mice and humans [21, 24]. The predicted product is a relatively small membrane protein (231 amino acids) containing a highly conserved region within and around the second hydrophobic domain. Previous studies established that TMIE is required for MET in hair cells [22, 26, 27] and is an integral member of the complex [26]. How TMIE contributes to the function of the MET complex was not clear. Our comprehensive structure-function analysis of Tmie revealed that the functional capacity of various *tmie* mutant constructs is determined by their efficacy in localizing Tmc2b-GFP to the hair bundle, as summarized in Fig 9A and modeled in Fig 9B. These findings unveil a hitherto unexpected role for Tmie in promoting the localization of the channel subunits Tmc1 and Tmc2b to the site of MET. Our study broadens our understanding of the assembly of the MET complex and points to a pivotal role of Tmie in this process.

A previous report on the *ru1000* mutant suggested that Tmie’s role in zebrafish hair cells was developmental, with mutant lateral line hair cells showing stunted kinocilia and the absence of tip links [22]. In our hands we did not observe any gross morphological defects *in vivo* or at the ultrastructural level in the inner ear, and the localization pattern and levels of Pcdh15a and Lhfpl5a were unaffected in *tmie*^*ru1000*^ larvae. This observation is consistent with intact hair-bundle morphology. In contrast, splayed stereocilia are a dominant feature of hair cells missing their tip links such as *pcdh15a* or *lhfpl5a* mutants [18, 33]. In TMIE-deficient mice, hair cell morphology is also grossly normal up to P7 [26, 27]. In agreement with a previous study in mice [26], our results indicate that *tmie*^*ru1000*^ mutants are profoundly deaf due to ablation of MET in hair cells. We were able to fully rescue this deficit in zebrafish *ru1000* mutants by exogenous expression of a GFP-tagged transgene of *tmie*. Exogenous expression gave rise to variable levels of Tmie-GFP in hair bundles, with lower levels revealing a punctate pattern expected for a member of the MET complex, and higher expression levels leading to expression throughout the stereocilia. Excess Tmie-GFP did not appear to cause adverse effects in hair cells, which is consistent with a previous study in the *circler* mouse mutant [39].

### Trafficking patterns of Tmie and Tmcs suggests trafficking ‘cliques’ of MET proteins

There are still many open questions regarding the sequence of events for assembly of the MET complex. One question is whether assembly occurs before or after ascension of stereocilia. Previous reports demonstrate that bundle targeting of Pcdh15a relies upon the presence of Lhfpl5a [18, 19, 40]. Likewise, LHFPL5 requires PCDH15 for localization at stereociliary tips [19, 40]. Here, we demonstrate that Tmc1 and 2b require Tmie for targeting to the bundle, while Pcdh15a and Lhfpl5a are unaffected in *tmie* mutants. Collectively, these findings suggest that Pcdh15a-Lhfpl5a traffick together in one unit, and Tmie-Tmcs traffick in a separate unit. This conclusion supports the idea that the MET complex proteins traffick to stereocilia in multiple groups and then assemble the full MET complex at the site of transduction.

Another interesting finding from our data is that Tmie may be the exception to the rule of co-dependent transport to the hair bundle because it retains normal trafficking patterns in the absence of individual MET proteins (Pcdh15a, Lhfpl5a, or Tmc1/2a/2b). As our experiment used an overexpressed Tmie transgene, measurement of endogenous levels of Tmie in other MET mutants would be an important follow-up experiment for future studies.

### Tmie has distinct regions required for localization and function

Using our transgenic *tmie* constructs, we identified specific regions of Tmie that are required for trafficking or function (Fig 9). Despite reduced bundle targeting, a truncated version of Tmie containing amino acids 1–138 showed full functional rescue, though only when it was overexpressed as a transgene (S3 Fig). We speculate that higher levels of expression enabled this truncated version to overcome inefficient trafficking. Conversely, despite normal localization to the bundle, expression of the Δ97–113 construct did not rescue function at all, even when it was likewise overexpressed. These results demonstrate that Tmie’s functional role is separate from its ability to target to the bundle.

In total, three constructs resulted in reduced targeting of Tmie to the bundle, namely SP63-231, CD8-2TM, and the 1–138 construct, the last of which truncates the C-terminus. As the predicted topology of Tmie places the C-terminus on the cytoplasmic side of a vesicle, this topology would expose amino acids 97–231 for recognition by trafficking machinery. Alteration of the C-terminus results in partial or full mistargeting of Tmie. We suspected a potential bundle targeting signal in amino acids 139–231. However, when we express this peptide (*139-231-GFP*), fluorescence is diffused throughout the cell, including the kinocilium and nucleus. These results suggest that while the C-terminus is integral to bundle targeting, there are other regions of Tmie that are required for proper stereocilia localization. These regions include a portion of the external peptide sequence and residues in the 2TM domain of Tmie (Fig 9B, blue outline). Reduced efficiency in bundle targeting may be due to impaired interactions with externally localized components of the MET complex or partial misfolding. This idea is supported by the finding that expression of constructs SP63-231 and 2TM-CD8 lead to impaired rescue of function in *tmie*^*ru1000*^ larvae, while the partially mislocalized truncation construct (1–138) showed full functional rescue when overexpressed as a transgene.

### Tmie promotes the levels of Tmc1/2b in the hair bundle

The regulatory role of Tmie with respect to the Tmcs is strongly supported by the strikingly different effects of loss of Tmie versus overexpression of Tmie. When Tmie is absent from the bundles, so are Tmc 1 and 2b; when Tmie is overexpressed, the bundle levels of Tmc1 and 2b are boosted as well. These results disagree with a previous finding in mice showing that Myc-TMC2 is present in hair bundles of TMIE-deficient cochlear hair cells [26]. This discrepancy may be due to different methods of expression, different hair cell types, or species-specific effects. Zhao et al. used a cytomegalovirus promoter to drive high levels of expression of Myc-TMC2 in an *in vitro* explant of cochlear tissue. Vestibular hair cells were not characterized, and the effects of TMIE-deficiency on localization of TMC1, which is the dominant TMC protein in mature cochlear hair cells, was not reported in their study. Further investigation is warranted to determine if the Tmie-Tmc relationship uncovered by our experiments is a conserved feature or is potentially dependent on the type of hair cell, as MET components may vary among different cell types.

One important question is whether Tmie and the Tmcs can physically interact to form a complex that is transported to the hair bundle. A direct interaction of the mouse TMC1/2 and TMIE proteins was not detected in a heterologous system [26]. However, our *in vivo* analysis suggests a strong dependency of the Tmcs on Tmie. There may be an indirect interaction, or an as-yet-undetected direct interaction between the Tmcs and Tmie, perhaps missed by previous experiments because the native hair cell environment is essential for the interaction to occur. When mammalian TMCs are expressed in heterologous cell types, they mainly populate inner membrane compartments such as the endoplasmic reticulum [8, 10, 11, 15, 26]. Successful folding and trafficking of the TMCs to the plasma membrane may require specialized trafficking components in the hair-cell secretory pathway. One example of such as factor is the Golgi-enriched Tomt protein, which is essential for Tmc1/2 trafficking in hair cells [11, 36]; there are likely to be other components as well.

### Experimental support for cleavage of Tmie’s first hydrophobic region

While the membrane topology of Tmie has not been biochemically determined, *Phobius* software predicts an N-terminal signal peptide in mouse and human TMIE, and a transmembrane helix in zebrafish Tmie [41]. Interestingly, the orthologues in *C*. *elegans* and *D*. *melanogaster* do not contain this first hydrophobic region of Tmie. Upon removal of this region from zebrafish Tmie (construct *SP44-231*), we observed a localization pattern identical to full-length Tmie and full functional rescue of *tmie*-deficient fish. In addition, expressing the SP44-231 construct in *tmie*^*ru1000*^ larvae rescues Tmc2b-GFP bundle expression to wild type levels or higher. To our knowledge, these results are the first *in vivo* evidence that vertebrate Tmie can function without the first hydrophobic domain. Our study supports the notion that Tmie undergoes cleavage, resulting in a single-pass membrane protein that functions in the MET complex (Fig 9B).

### The 2TM and intracellular neighboring domain may mediate integration within the MET complex

Only two of our Tmie constructs displayed dominant negative effects in wild type larvae (*SP63-231* and *Δ63–73*), suggesting successful integration into the MET complex and competition or interference with endogenous Tmie. Both of these constructs delete unique parts of the extracellular region of Tmie, and have little to no rescue of mechanosensitivity in *tmie*^*ru1000*^ mutants. The transmembrane chimeras in our study also yield impaired rescue but do not appear to affect the function of endogenous Tmie in wild-type hair cells. These data suggest that the entire 2TM domain is required to produce the dominant negative effect on endogenous Tmie. Combined with the finding that replacement of the 2TM with an unrelated helix causes instability of Tmie in mature hair cells, we propose that the 2TM is essential for integration of Tmie into the MET complex.

One construct does not fit this hypothesis: Δ97–113, which contains the full 2TM and has no functional rescue, but does not have a dominant negative effect. However, this region contains arginine residues that have previously been implicated in human deafness [24, 42–44] and that in mice were linked to interactions with PCDH15-CD2 [26]. It is possible that the cytoplasmic region adjacent to the 2TM is also required for full integration into the MET complex, perhaps through interactions unrelated to the Tmcs. Interestingly, one of the mouse mutations, R93W, resulted in loss of TMIE localization at the site of MET in cochlear hair cells. In contrast to these findings, when we remove this entire intracellular region from zebrafish Tmie, it is still capable of targeting to hair bundles. This result may reflect different targeting motifs for different hair-cell types or species differences in recognition sequences for trafficking machinery.

### Tmie stabilizes Tmc expression at the site of MET

When co-expressed with Tmc2b-GFP, our Tmie constructs reveal a strong link between function and Tmc bundle expression. In addition to defects in targeting Tmcs to the hair bundle (constructs *Δ97–113* and *CD8-2TM*), our data also suggest a role for Tmie in maintaining the levels of Tmc2b in stereocilia. We previously reported that trafficking and stability/maintenance are two distinct events that can be separated experimentally by examining transgene expression in immature vs mature bundles [18]. The only region of Tmie with a clear effect on maintenance of bundle Tmc signal was amino acids 63–73. Loss of these residues resulted in Tmc2b-GFP signal in immature hair cells suggesting proper trafficking, but a large reduction in mature cells suggesting poor maintenance in the MET complex. Based on our data, we conclude that the first half of the transmembrane domain and the intracellular residues 97–113 are required for targeting the Tmc subunits to the site of MET (Fig 9B, yellow fill), while the extracellular residues 63–73 stabilize Tmc expression in the MET complex (Fig 9B, orange fill). Since co-expression of Tmc2b-GFP can overcome the functional deficits in constructs *SP63-231* and *2TM-CD8*, we propose that residues 44–62 and the second half of the 2TM are important but not absolutely essential to regulating Tmc bundle expression. This finding reinforces the significance of our data obtained with the constructs *Δ63–73*, *CD8-2TM*, and *Δ97–113*, which still fail to rescue Tmc2b-GFP levels. In addition, we demonstrated that CD8-2TM also does not rescue expression of Tmc1-GFP, suggesting that similar mechanisms are employed for trafficking of both Tmc1 and Tmc2 members of the Tmc superfamily.

In sum, through a systematic *in vivo* analysis of *tmie* via transgenic expression, we identified new functional domains of Tmie. We demonstrated a strong link between Tmie’s function and Tmc1/2b expression in the bundle. Evidence continues to mount that the Tmcs are pore-forming subunits of the MET channel, and our results implicate Tmie in promoting and maintaining the localization of the MET channel. The precise mechanism underlying Tmie’s regulation of the Tmcs awaits further investigation.

## Materials and methods

### Ethics statement

Animal research was in compliance with guidelines from the Institutional Animal Care and Use Committee at Oregon Health and Science University (protocol # IP00000100).

### Zebrafish husbandry

We maintained zebrafish (*Danio rerio*, txid7955) at 28°C and bred according to standard conditions. In this study, we used the following zebrafish mutant lines: *tmie*^*ru1000*^ [22], *tmie*^*t26171*^, *pcdh15a*^*psi7*^ [10], *lhfpl5a*^*tm290d*^ [45], and *tomt*^*tk256c*^ [11]. We maintained all zebrafish lines in a Tübingen or Top long fin wild type background. We examined larvae at 3–7 days post-fertilization (dpf), of undifferentiated sex. For experiments involving single transgenes, non-transgenic heterozygotes were crossed to transgenic fish in the homozygous or heterozygous mutant background. We genotyped larvae by PCR and subsequent digestion or DNA sequencing, or by behavior phenotype prior to experiments, as detailed for each experiment below. Primers are listed in Table 1.

**Table 1:** List of primers used in this study.

*Primers for plasmid construction*			
Plasmid		Forward (5’—3’)	Reverse (5’—3’)
pME-Tmie		GGGGACAAGTTTGTACAAAAAAGCAGGCTCCAAACATGAGACGCGGGAGAAGAA	GGGGACCACTTTGTACAAGAAAGCTGGGTCTTTCTTCGCAGGCTTCTTGG
pME-SP44-231		GGGGACAAGTTTGTACAAAAAAGCAGGCTCCAAACATGATTTTGTCGGGTCTCCTTTTACCCGCGTTATGGGGACTGGCGCTCGGCCAGATACCAGACCCAGAGCT	same as pME-Tmie
pME-SP63-231		ACCTCAGAAACAGTGGTGTTTTGGGGA	GCCGAGCGCCAGTCCC
pME-Δ63–73		TTATGGCAGGTTGTGGGCATTTTC	GACGGGGTCTGGCTTTTTCG
pME-CD8	PCR 1	same as pME-Tmie	GAGAAGGACCCCACAAGTCCCGGCCAGGGGCGCCCAGATGTATCGAAGTCCCCAAAACAC
	PCR 2	TTGTGGGGTCCTTCTCCTGTCACTGGTTATCACCCTTTACTGCAAATGCCGAATCCCAC	same as pME-Tmie
pME-CD8-2TM	PCR 1	ATGAGACGCGGGAGAAGAAGAGGGAAAATG	CCCACAAGTCCCGGCCAGGGGCGCCCAGATGTATCGAAGTCCCCAAAACACCACTGTTTC
	PCR 2	TGGCCGGGACTTGTGGGATCTTAGCAATAATAATTACGCTCTGCTGCATCTTCAAATGCC	TTTCTTCGCAGGCTTCTTGGCACCTC
pME-2TM-CD8	PCR 1	same as pME-CD8-2TM	AAAGGGTGATAACCAGTGACAGGAGAAGGACGAACATGGAGAAAATGCCCACAACCTGCC
	PCR 2	CCTTCTCCTGTCACTGGTTATCACCCTTTACTGCAAATGCCGAATCCCACGGACG	same as pME-CD8-2TM
pME-Δ97–113		AGACTTGCTGCGAAAAATTATGCCAAC	GAAGATGCAGCAGAGCGTAATTATTATTGC
pME-Δ114–138		GCGGCAAAGGTTGAGGTGAAG	TTGCGCGTGCCGAGC
pME-1-138		same as pME-Tmie	GGGGACCACTTTGTACAAGAAAGCTGGGTCGCCGGGCACCTCAG
pME-1-113		same as pME-Tmie	GGGGACCACTTTGTACAAGAAAGCTGGGTCTTGCGCGTGCCGAG
pME-Tmie-short		same as pME-Tmie	GGGGACCACTTTGTACAAGAAAGCTGGGTCAGTGCCAGGATTGGCTG
pME-139-231		GGGGACAAGTTTGTACAAAAAAGCAGGCTCCAAACATGGCGGCAAAGGTTGAGGT	same as pME-Tmie
*Primers for RT-PCR and semi-quantitative PCR*			
To amplify:		Forward (5’—3’)	Reverse (5’—3’)
*t26171* cDNA		ATATGCCAACACATTGGAGACGGTGC	CCCTGAGGTGTGTGTGAGTGTTCA
*tmie-short*		ATGAGACGCCCCAGAAGAAGAGGGAAAATGGCGATG	TTAAGTGCCAGGATTGGCCGGTTCATCTTCTTCCCTG
*tmie*		GGTGCAAAAGAAGGTAAAGAAGAGG	GCTTCTTTCTTTGGTGCTCCT
*gapdh*		ATCATCTCTGCCCCAAGTGC	TGCAGGAGGCATTGCTTACA
*Primers for identifying mutants*			
Mutant		Forward (5’—3’)	Reverse (5’—3’)
*ru1000*		TGTTTCGTCCAGGCTGAAG	GGCCTCATAAAACACAAGCA
*psi7*		TTGGCACCACTATCTTTACCG	ACAGAAGGCACCTGGAAAAC
*tm290d*		TGGTCTTCATCCAGCCCTAC	CGATCAGCAGCAAAGAGATG
*tk256c*		TGTGTATTGCAGGTCAGTGTTG	AAGCGTTTTTCTGGGTGTTG
*t26171*		GCACAGCCCTAATGGATACAG	GCTTCTTTCTTTGGTGCTCCT

### Gene accession numbers for mutants and transgenes

*tmie* (accession no. F1QA80), *tmc1* (accession no. F1QFU0), *tmc2b* (accession no. F1QZE9), *tomt* (accession no. A0A193KX02), *pcdh15a* (accession no. Q5ICW6), *lhfpl5a* (accession no. F1Q837), *actba* (accession no. Q7ZVI7).

### Transgenic lines and plasmid construction

The following previously published transgenic lines were used: *Tg(-6myo6b*:*β-actin-GFP-pA)* [34], *Tg(-6myo6b*:*pcdh15aCD3-mEGFP-pA)* [18], and *Tg(-6myo6b*:*GFP-lhfpl5a-pA)*, *Tg(-6myo6b*:*Tmc1-mEGFP-pA)*, *Tg(-6myo6b*:*Tmc2b-mEGFP-pA)* [11].

To generate the *tmie* expression vectors, we used the Tol2/Gateway system [46]. The pDestination vector contained either a *cmlc2*:*GFP* heart marker or α-*ACry*:*mCherry* eye marker for sorting. pDESTtol2pACrymCherry was a gift from Joachim Berger and Peter Currie (Addgene plasmid # 64023, [47]).

The 5’ entry vector contained the promoter for the *myosin 6b* gene, which drives expression only in hair cells. All *tmie* transgenic constructs were subcloned into the middle entry vector using PCR or bridging PCR and confirmed by sequencing. The primers for each vector are listed in Table 1. For GFP-tagging, we used a 3’ entry vector with a flexible linker (GHGTGSTGSGSS) followed by *mEGFP*. For *NLS(mCherry)* experiments, a p2A self-cleaving peptide (GSGATNFSLLKQAGDVEENPGP) was interposed between the *tmie* construct and the *NLS(mCherry)*. This causes translation of a fusion protein that is subsequently cleaved into the two final proteins. The 2TM helix replacements from residues 21–43 result in the following chimeric helices: CD8 (YIWAPLAGTCGVLLLSLVITLYC), CD8-2TM (YIWAPLAGTCGILAIIITLCCIF), and 2TM-CD8 (LWQVVGIFSMFVLLLSLVITLYC).

Multisite Gateway LR reactions [48, 49] were performed to generate the following constructs: *pDest(-6myo6b*:*tmie-GFP-pA)*, *pDest(-6myo6b*:*tmie-short-GFP-pA)*, *pDest(-6myo6b*:*SP44-231-GFP-pA)*, *pDest(-6myo6b*:*SP63-231-GFP-pA)*, *pDest(-6myo6b*:*Δ63-73-GFP-pA)*, *pDest(-6myo6b*:*CD8-GFP-pA)*, *pDest(-6myo6b*:*CD8-2TM-GFP-pA)*, *pDest(-6myo6b*:*2TM-CD8-GFP-pA)*, *pDest(-6myo6b*:*Δ97-113-GFP-pA)*, *pDest(-6myo6b*:*Δ114-138-GFP-pA)*, *pDest(-6myo6b*:*1-113-GFP-pA)*, *pDest(-6myo6b*:*1-138-GFP-pA)*, *pDest(-6myo6b*:*tmie-p2A-NLS(mCherry)-pA)*, *pDest(-6myo6b*:*SP63-231-p2A-NLS(mCherry)-pA)*, *pDest(-6myo6b*:*Δ63-73-p2A-NLS(mCherry)-pA)*, *pDest(-6myo6b*:*CD8-2TM-p2A-NLS(mCherry)-pA)*, *pDest(-6myo6b*:*Δ97-113-p2A-NLS(mCherry)-pA)*.

To generate transgenic fish, plasmid DNA and *tol2* transposase mRNA were co-injected into single-cell fertilized eggs, as previously described (Kwan et al., 2007). For each construct, 200+ eggs from an incross of *tmie*^*ru1000*^ heterozygotes were injected. To obtain stable transgenic lines, >24 larvae with strong marker expression were raised as potential founders. For each GFP-tagged transgene, at least two founder lines were generated and examined for visible bundle expression. To equalize expression of each transgene within a clutch, for each *tmie* construct we isolated a line with a transgene transmission rate of 50%, indicating single transgene insertion. The *CD8-2TM-GFP* construct was the exception; we instead identified a single adult with mosaic transmission of the transgene (transmitted to >10% of offspring) and used this line in FM and microphonics experiments. Imaging during the FM experiment confirmed that CD8-2TM-GFP was consistently highly expressed (Fig 6A, *CD8-2TM*). For *NLS(mCherry)* experiments, injected fish were raised to adulthood and genotyped to identify *tmie*^*ru1000*^ heterozygotes and homozygotes. We identified founders for each construct and then crossed these founders to *tmie*^*ru1000*^ heterozygotes carrying *Tg(myo6b*:*tmc2b-GFP)*. This generated offspring that expressed both transgenes in the *tmie*^*ru1000*^ mutant background, and we used these larvae for experiments. In *SP44-231*, *SP63-231*, and *CD8-2TM*, and full-length *tmie* in the Tmc1-GFP background, stable transgenic lines were generated from the founder before experiments were carried out.

### Microscopy

We anesthetized live larvae with E3 plus 0.03% 3-amino benzoic acid ethylester (MESAB; Western Chemical) and mounted in 1.5% low-melting-point agarose (Sigma-Aldrich cas. # 39346-81-1), with the exception of the morphology images from Figs 1A and 7A in which larvae were pinned with glass rods and imaged in E3 or extracellular solution containing MESAB. We captured the image in Fig 7A at room temperature using a Hamamatsu digital camera (C11440, ORCA-flash2.8), MetaMorph Advanced NX software, and an upright Leica DMLFS microscope. We used differential interference contrast (DIC) with a Leica HC PL Fluotar 10x/0.3 lens. For all imaging except Fig 7A, images were captured at room temperature using an Axiocam MrM camera, Zeiss Zen software, and an upright Zeiss LSM700 laser-scanning confocal microscope. We used DIC with one of two water-immersion lenses: Plan Apochromat 40x/1.0 DIC, or Acroplan 63x/0.95 W. Laser power and gain were unique for each fluorophore to prevent photobleaching. We averaged 2x or 4x for each image, consistent within each experiment. The Tmc1-GFP and Tmc2b-GFP transgenes are very dim, and high laser power (4%) and gain (1100) were necessary to detect signal in wild types. At these settings, autofluorescence from other wavelengths can falsely enhance the emission peak at 488. To reduce detection of autofluorescence, we simultaneously collected light on a second channel with an emission peak at 640 nm.

### Transmission electron microscopy (TEM)

We sorted 5 dpf zebrafish larvae by behavior (tap sensitivity and balance), then fixed them overnight at 4°C in PBS containing fresh 1% EM-grade formaldehyde (Electron Microscopy Sciences, Hatfield, PA) and fresh 2% EM-grade glutaraldehyde (Tousimis Research Corporation, Cat # 1060A). For further fixation and contrast, we incubated larvae for 10 min on ice with 1% osmium tetroxide (Electron Microscopy Sciences, Hatfield, PA), followed by 1 hr on ice in 1% uranyl acetate (Electron Microscopy Sciences, Hatfield, PA). We dehydrated larvae in a graded series of EM-grade acetone (Electron Microscopy Sciences, Hatfield, PA), then embedded in Embed-812 (Electron Microscopy Sciences, Hatfield, PA). We collected thin sections on PELCO 200 mesh nickel grids (Ted Pella, Redding, CA), and stained with 4% uranyl acetate and Reynolds lead citrate. We collected electron microscopy images on an FEI Tecnai 12 BioTWIN transmission electron microscope (ThermoFisher Scientific, Hillsboro, OR) operated at an 80 kV accelerating voltage.

### Auditory evoked behavioral response (AEBR)

Experiments were conducted as previously described [50]. Briefly, 6 dpf larvae were placed in six central wells of a 96-well microplate mounted on an audio speaker. Pure tones were played every 15 s for 3 min (twelve 100 ms stimuli at 1 kHz, sound pressure level 157 dB, denoted by asterisks in Fig 1B). Responses were recorded in the dark inside a Zebrabox monitoring system (ViewPoint Life Sciences). Peaks represent pixel changes from larval movement. A response was considered positive if it occurred within two seconds after the stimulus and surpassed threshold to be considered evoked, not spontaneous (Fig 1B, green indicates movement detected, magenta indicates threshold surpassed). For each larva, we used the best response rate out of three trials. Response was quantified by dividing the number of positive responses by total stimuli (12) and converting to a percent. If the larvae moved within two seconds before a stimulus, that stimulus was dropped from the trial data set (i.e. the number of total stimuli would become 11). Each data point on the graph in Fig 1C is the percent response of an individual larva. We used a two-tailed unpaired t-test with Welch’s correction to determine significance, ****p < 0.0001. Wild type and mutant larvae were genotyped by FM 1–43 labeling.

### Immunofluorescent staining

We used an anti-Pcdh5a monoclonal antibody directed against amino acids 1–324 [10] as described previously [18]. In brief, we sorted wild type and *ru1000* larvae at 5 dpf by behavior (tap response and balance). We then fixed 8 larvae per 2ml microtube in Phosphate Buffered Saline + 0.01% Tween-20 (PBST) + 4% paraformaldehyde, rotating overnight on a nutator at 4°C. We washed with PBST 3x for 10 min each, then permeabilized with PBS + 0.5% TritonX100 on a shaking table (50 rpm) for 1 hour, then at 4°C overnight without shaking. We blocked with PBS + 1% DMSO + 5% goat serum + 1% Bovine Serum Albumen (BSA, Sigma-Aldrich Lot # SLSF5374V) for 2 hours minimum at room temperature on a shaking table (50 rpm). We applied the mouse anti-1C4 Pcdh15a antibody at 1:200 in blocking buffer overnight on the nutator at 4°C. We washed with PBST 3x for 15 minutes each on the shaking table (50 rpm). We applied blocking buffer + secondary antibody, 546-conjugated goat anti-mouse IgG at 1:500 concentration (Life Technologies), and also included phalloidin-488 at 1:100 to visualize Beta-actin filaments in hair bundles, on a shaking table (50 rpm) for 4–5 hours in the dark at room temperature. We washed with PBST 3x for 10 minutes each and stored at 4°C before imaging.

### cDNA generation by Reverse Transcription Polymerase Chain Reaction (RT-PCR) and semi-quantitative PCR

For S3 Fig and Fig 4A, we extracted total RNA using the RNeasy mini kit (Qiagen). Larvae were homogenized using a 25 gauge syringe (Becton Dickinson, ref # 309626). To reverse transcribe cDNA we used the RNA to cDNA EcoDry Premix (Clontech, Cat # 639549). We then performed PCR on the cDNA using High Fidelity Phusion polymerase (New England Biolabs, Cat # M0530). To amplify the short isoform of Tmie (*Tmie-short*) and the *t26171* allele, we sorted 30 wild type and 30 *t26171* larvae by behavior (tap sensitivity and balance defect) at 5 dpf and used the pooled cDNA as template for the PCR reactions. Primers are listed in Table 1. Both transcripts were verified by DNA sequencing.

For Fig 4A, we sorted non-transgenic wild type (hetereozygote) and *tmie*^*ru1000*^ (homozygote) larvae by behavior at 5 dpf; the transgenic pool contained a mix of *tmie*^*ru1000*^ hetereozygotes and homozygotes (no behavior difference because full-length *tmie* transgene rescued phenotype). The *tmie* transgene was a single insert with low mCherry expression, the same line used for the data in Fig 4B and 4C, and S5 Fig, *Tmie*. For each genotype, n = 20 larvae were homogenized. We performed PCR at 30, 35, and 40 cycles. We ran on 2% gel and loaded 25 ul of *tmie* PCR product; we loaded only 10 ul of *gapdh* product to avoid saturating the bands. We quantified the 40 cycle bands in ImageJ using *gapdh* levels to normalize *tmie* levels. Primers are listed in Table 1; both *tmie* and *gapdh* produced 98 bp amplicons. The primers for *tmie* amplified a region within exon 4 in order to detect transcripts from both transgene and endogenous *tmie*.

### FM 1–43 and FM 4–64 labeling

Larvae were briefly exposed to E3 containing either 3μM N-(3-Triethylammoniumpropyl)-4-(4-(Dibutylamino)styryl)Pyridinium Dibromide (FM 1–43, Life Technologies) or 3μM of the red-shifted *N[scap]*-(3-triethylammoniumpropyl)-4-(6-(4-(diethylamino)phenyl)hexatrienyl)pyridinium dibromide (FM4-64; Invitrogen). After exposure for 25–30 seconds, larvae were washed 3x in E3 and neuromasts were imaged from top-down. Neuromasts were chosen based upon their orientation, bundles pointing upward preferred. Typically, three neuromasts were examined per larvae, from the head or trunk depending on best angle; we never observed differences in posterior/anterior labeling. Laser power was adjusted for each experiment to avoid saturation of pixels but was consistent within a clutch. FM levels were quantified in ImageJ [51] as described previously [10]. In brief, maximum projections of each neuromast were generated using seven optical sections, beginning at the cuticular plate and moving down through the soma (magenta bracket, Fig 1G). We then measured the integrated density of the channel with an emission peak at 640 nm for FM 4–64, and at 488 nm for FM 1–43. This integrated density value was divided by the number of cells, thus converting each neuromast into a single plot point of integrated density per cell (IntDens/cell). Statistical analyses were always performed between direct siblings. For Fig 6, individual values were divided by the mean of the sibling wild type neuromasts in order to display the data as a percent of wild type, making it easier to compare across groups. Statistical significance was determined within an individual clutch using one-way ANOVA. We PCR-genotyped all larvae from the *Tmie-GFP* line; after confirming results, for all other *tmie* construct experiments, we continued to genotype transgenic larvae by PCR but genotyped non-transgenic wild type and *tmie*^*ru1000*^ larvae by behavior and expected FM labeling patterns.

### Microphonics

Larvae at 3 dpf were anesthetized in extracellular solution (140mM NaCl, 2mM KCl, 2mM CaCl_2_, 1mM MgCl_2_, and 10mM 4-(2-hydroxyethyl)-1-piperazineethanesulfonic acid (HEPES); pH 7.4) containing 0.02% 3-amino benzoic acid ethylester (MESAB; Western Chemical). Two glass fibers straddled the yolk to pin the larvae against a perpendicular cross-fiber. Recording pipettes were pulled from borosilicate glass with filament, O.D.: 1.5 mm, O.D.: 0.86 mm, 10 cm length (Sutter, item # BF150-86-10, fire polished). Using the Sutter Puller (model P-97), we pulled the pipettes into a long shank with a resistance of 10-20MΩ. We then used a Sutter Beveler with impedance meter (model BV-10C) to bevel the edges of the recording pipettes to a resistance of 3–6 MΩ. We pulled a second pipette to a long shank and fire polished to a closed bulb, and then attached this rod to a piezo actuator (shielded with tin foil). The rod was then pressed to the front of the head behind the lower eye, level with the otoliths in the ear of interest, to hold the head in place while the recording pipette was advanced until it pierced the inner ear cover. Although it has been demonstrated that size of response is unchanged by entry point [52], we maintained a consistent entry point dorsal to the anterior crista and lateral to the posterior crista (see Fig 7A). After the recording pipette was situated, the piezo pipette was then moved back to a position in light contact with the head. We drove the piezo with a High Power Amplifier (piezosystem jena, System ENT/ENV, serial # E18605), and recorded responses in current clamp mode with a patch-clamp amplifier (HEKA, EPC 10 usb double, serial # 550089). Each stimulus was of 20 ms duration, with 20 ms pre- and post-stimulus periods. We used either a sine wave or a voltage step and recorded at 20 kHz, collecting 200 traces per experiment. In Fig 1H, we used a 200 Hz sine wave at 10V, based on reports that 200 Hz elicited the strongest response [38]. In Fig 7, we used multiple step stimuli at varying voltages (2V, 3V, 4V, 5V, 6V, and 10V). The piezo signal was low-pass filtered at 500Hz using the Low-Pass Bessel Filter 8 Pole (Warner Instruments). Microphonic potential responses were amplified 1000x and filtered between 0.1–3000 Hz by the Brownlee Precision Instrumentation Amplifier (Model 440). We used Igor Pro for analysis. We averaged each set of 200 traces to generate one trace response per fish, then measured baseline-to-peak amplitude. These amplitudes were used to generate the graphs in Fig 7. Statistical significance was determined by 2-way ANOVA comparing all groups to wild type non-transgenic siblings. We used PCR to genotype larvae.

### Quantification of fluorescence signal in the ROI

Using ImageJ, maximum projections of each crista were generated for analysis (5 sections per stack for Tmc1-GFP in Fig 3D and Tmc2b-GFP in Fig 3F, and 13 sections per stack for Tmc1-GFP in Fig 4C and Tmc2b-GFP in Fig 4E. Quantification of Tmc-GFP bundle fluorescence was achieved by outlining each bundle to encompass the entire region of interest (ROI) in a single hand-drawn area (Fig 8A, right panel, black outline). For S2 Fig, the ROI was the soma region of the crista or the entire cell including soma and bundle (max projection of 13 sections). For S5 Fig, the ROI was the soma region containing nuclei of hair cells. In the ROI, we quantified the integrated density of the channel with an emission peak at 480 nm for GFP-tagged constructs, and with an emission peak at 640nm for mCherry. For GFP-tagged constructs, this was repeated in the region above the bundles containing only inner ear fluid and the kinocilia in order to subtract background fluorescence. Background for nuclear mCherry was measured from the soma region between the bundle and nuclei. Each lateral crista generated one data point in all quantification graphs. In some cases, we saw single cells that appeared to have a GFP-fill, probably due to clipping of the GFP tag. We excluded these cells from analyses, since they falsely increased the signal. Likewise, for quantification of soma GFP, we excluded crista with immature hair cells that highly expressed the *myo6b* driven transgene. Due to the 3D nature of the mound-shaped cristae, it was difficult to completely exclude the apical soma region, leading the bundle signals to average above zero in *tmie*^*ru1000*^ expressing either Tmc1-GFP or Tmc2b-GFP. For determination of significance, we used the Kruskal-Wallis test for quantification of Tmc2b-GFP bundle signal in the background of co-expressed SP44-231, SP63-231, and 2TM-CD8; all others quantifications with three or more groups are one-way ANOVA. For comparison of two groups, we used two-tailed unpaired t-tests with Welch’s correction. We PCR-genotyped all larvae from Fig 3B–3J; when introducing co-expression of *tmie* constructs, we continued to PCR-genotype larvae containing the *tmie*-transgene but genotyped non-*tmie*-transgene wild type and *tmie*^*ru1000*^ larvae by behavior.

### Statistical power

In experiments subjected to quantitative analysis, we used G*power [53] to determine the sample size required. For microphonics, we used the strongest driver stimulus setting (10V) in these determinations.

## Supporting information

S1 Fig Tmie-GFP shows variable expression in stereocilia. Representative images of the lateral crista in a wild type larva at 6 dpf, captured using confocal microscopy. (A) The hair bundle region of hair cells expressing transgenic tmie-GFP driven by the myo6b promoter. The arrow and bracket show, respectively, the short kinocilium and stereocilia bundle of an immature hair cell. (B) A single hair bundle with “bundle fill” expression pattern produced by overexpression of Tmie-GFP. (C) A single bundle with Tmie-GFP concentrated along the beveled edge of the stereocilial staircase. (D) A single bundle with punctate expression of Tmie-GFP suggestive of localization at the tips of shorter stereocilia, the site of MET. Scale bar in (A) is 5μm, in (D) is 2μm.
(TIF)

S2 Fig Total cellular levels of expression of Tmc2b-GFP are not influenced by Tmie. (A-F) Plot of the integrated density of Tmc2b-GFP fluorescence in the ROI of lateral cristae from 4 dpf larvae. ROI in A and D is the soma region, in B and E is the whole hair cell, and in C and F is a subtraction of whole cell fluorescence minus soma fluorescence to roughly determine the relative contribution of bundle signal. Significance was determined by two-tailed unpaired t-test with Welch’s correction, **p < 0.01, ****p < 0.0001.
(TIF)

S3 Fig Differential effects on function with a genomic mutation and a transgene mimic. (A) Data for a novel mutant allele of *tmie*, *t26171*. DNA: Chromatographs of the DNA sequence of tmie in wild type (above) and *tmie*^*t26171*^ (below) showing the genomic region where the mutation occurs. An arginine is mutated to guanine in the splice acceptor (black box, above) of the final exon of *tmie*, exon 4. The dashed black box below indicates the mutated original splice acceptor site. Use of a cryptic splice acceptor (black box, below) 8 nucleotides downstream causes a frameshift and an early stop codon (*). cDNA: Chromatograph of the DNA sequence from RT-PCR of *tmie*^*t26171*^ larvae bridging exons 3 and 4. Protein: The predicted protein products, shown here as a two-pass transmembrane protein. The wild type protein has many charged residues (positive in light gray, negative in dark gray) that are lost in *tmie*^*t26171*^. Balance: Photos of wild type and *tmie*^*t26171*^ larvae, taken with a hand-held Canon camera. Arrow points to a larva that is upside-down, displaying a classic vestibular phenotype. (B) Top-down view of a representative neuromast after exposure to FM 4–64, imaged using confocal microscopy. The first panel is a single plane through the soma region while the second panel is a maximum projection of 7 panels through the soma region, beginning at the cuticular plate (as denoted by magenta bracket in Fig 1G). (C) Same as (B) except that the first panel shows the bundle region so that 1-138-GFP can be visualized in bundles (as depicted by dashed green line, Fig 1G). The transgene is driven by the *myo6b* promoter. (D) Plot of the integrated density of FM fluorescence per cell. We normalized values to the average of wild type siblings. Displayed wild type and *tmie*^*ru1000*^ data are from siblings of *Tg(1-138-GFP); tmie*^*ru1000*^ and are the same values reported in Fig 6. Data for *tmie*^*t26171*^ is from a separate experiment. Statistical significance determined by one-way ANOVA, ****p<0.0001. Scale bar is 10μm.
(TIF)

S4 Fig Expression pattern and functional rescue by *tmie* constructs CD8 and 139–231. All images were captured using confocal microscopy. (A) Stereocilia of a neuromast viewed from above. The same neuromast was imaged at 4 dpf and 6 dpf. In hair cells expressing CD8-GFP, signal was initially detected in immature bundles, but this expression was only detectable in soma by dpf 6 as the cells matured (n = 10 cells). (B) Maximum projection of neuromasts viewed from above; left panel shows only FM 4–64 while right panel adds CD8-GFP. No rescue of FM 4–64 labeling was observed in *tmie*^*ru1000*^ hair cells expressing CD8-GFP (n = 40 cells). (C) Maximum projection of the posterior crista in a *tmie*^*ru1000*^ larva with some hair cells expressing 139-231-GFP, which fills the cell (n = 43 cells). (D) Same as B except the transgene being expressed is 139-231-GFP. No rescue of FM 4–64 labeling was observed in *tmie*^*ru1000*^ hair cells expressing 139-231-GFP (n = 33 cells). Scale bars in A and C are 5μm, in B and D are 10μm.
(TIF)

S5 Fig Nuclear mCherry fluorescence does not correlate with GFP-tagged Tmc fluorescence. XY plots of the integrated density of nuclear mCherry fluorescence vs the integrated density of GFP-tagged Tmc fluorescence in the bundle region of lateral cristae. We examined 4 dpf larvae. (A) Bundle values for constructs CD8-2TM and Δ97–113 are the same as those reported in Fig 8H using co-expression with Tmc2b-GFP. Bundle values for the full-length Tmie construct are the same as those reported in Fig 4C using co-expression with Tmc1-GFP. (B) Bundle values are the same as those reported in Fig 8 using co-expression of each individual *tmie* construct with Tmc2b-GFP. We performed linear regressions to generate p-values.
(TIF)

S6 Fig Functional rescue of *tmie*^*ru1000*^ larvae by constructs SP63-231 and 2TM-CD8 is Tmc dose-dependent. (A) Mean amplitude of the response peak ± SD as a function of the stimulus intensity of the driver voltage, as described in Fig 7B. (B) XY plot of the amplitude of microphonic response vs the integrated density of Tmc2b-GFP fluorescence in the ROI. A 10V step stimulus was used to evoke microphonic potentials. The line is a linear regression with a Pearson r = 0.7222, p = 0.1682. (C) Same as A except with the *2TM-CD8* construct. (D) Same as B except with the *2TM-CD8* construct, r = 0.879, p = 0.0018. Significance determined by Pearson correlation coefficient. Measurements were from 4 dpf larvae; we used lateral cristae for imaging of Tmc2b-GFP.
(TIF)

S7 Fig Tmc2b-GFP targets to the bundle but does not maintain stable expression when co-expressed with the Δ63–73 construct of Tmie. Confocal images of single hair bundles from cells expressing transgenic Tmc2b-GFP driven by the *myo6b* promoter. Brackets show the stereocilia bundle, which is shorter in immature hair cells (white brackets) and longer in mature ones (black brackets). We examined lateral cristae from 4 dpf larvae. Scale bar is 2μm.
(TIF)

S8 Fig When co-expressed with CD8-2TM, Tmc1-GFP shows the same lack of bundle expression in *tmie*^*ru1000*^ as Tmc2b-GFP. Plot of the integrated density of Tmc1-GFP fluorescence in the ROI, expressed as arbitrary units. We examined lateral cristae from 4 dpf larvae. Significance was determined by one-way ANOVA, n ≥ 7, ****p < 0.0001.
(TIF)

S1 Data All data used for quantifications in this study. (XLSX)
