# Supplementary figures and images for: Subunits of the mechano-electrical transduction channel, Tmc1/2b, require Tmie to localize in zebrafish sensory hair cells
Source: PLoS Genet. 2019 Feb 6;15(2):e1007635. doi: 10.1371/journal.pgen.1007635 (PMC6380590; doi:10.1371/journal.pgen.1007635)

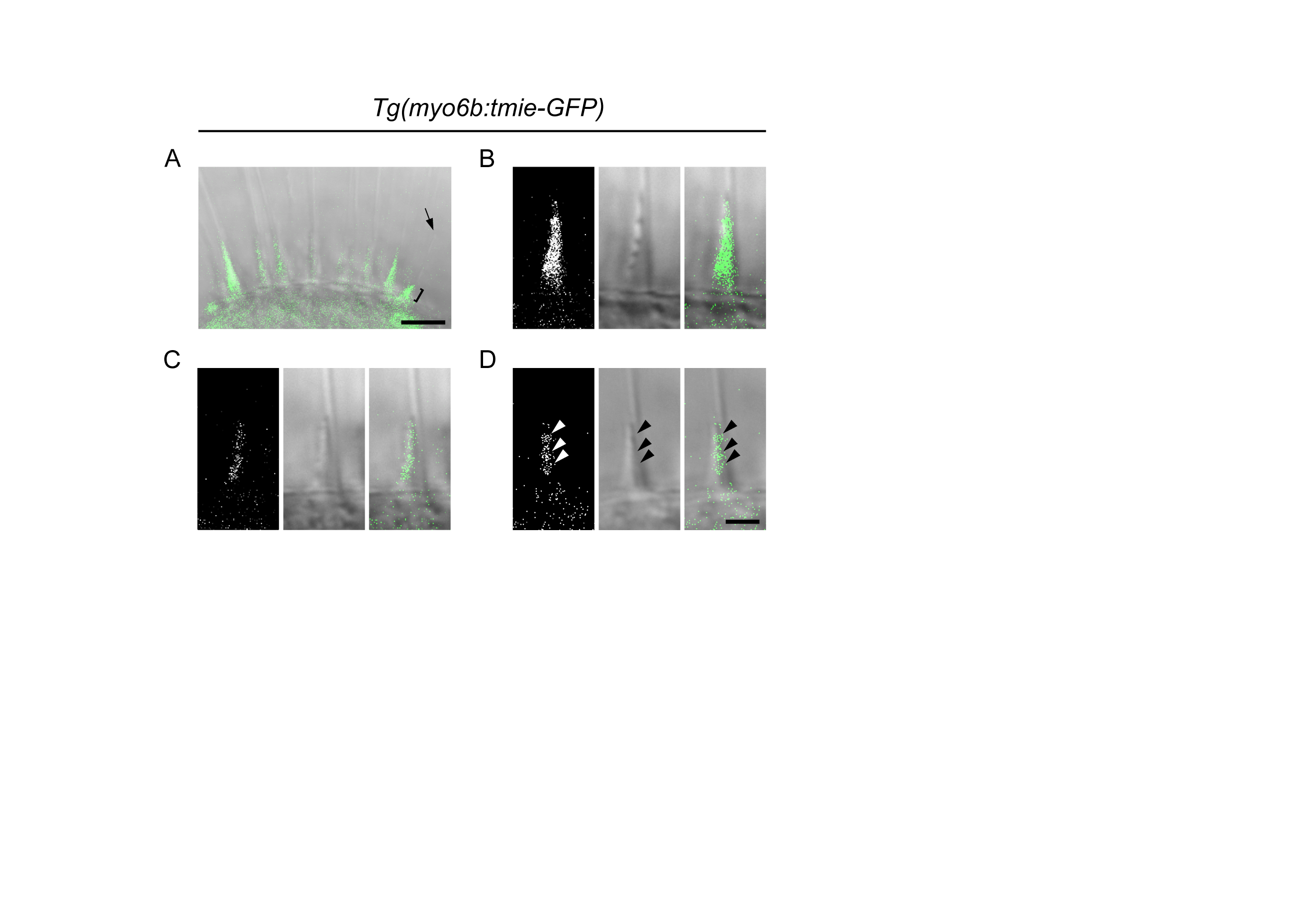

Supplement: S1 Fig — Representative images of the lateral crista in a wild type larva at 6 dpf, captured using confocal microscopy. (A) The hair bundle region of hair cells expressing transgenic tmie-GFP driven by the myo6b promoter. The arrow and bracket show, respectively, the short kinocilium and stereocilia bundle of an immature hair cell. (B) A single hair bundle with “bundle fill” expression pattern produced by overexpression of Tmie-GFP. (C) A single bundle with Tmie-GFP concentrated along the beveled edge of the stereocilial staircase. (D) A single bundle with punctate expression of Tmie-GFP suggestive of localization at the tips of shorter stereocilia, the site of MET. Scale bar in (A) is 5μm, in (D) is 2μm. (TIF) [file pgen.1007635.s001.tif]

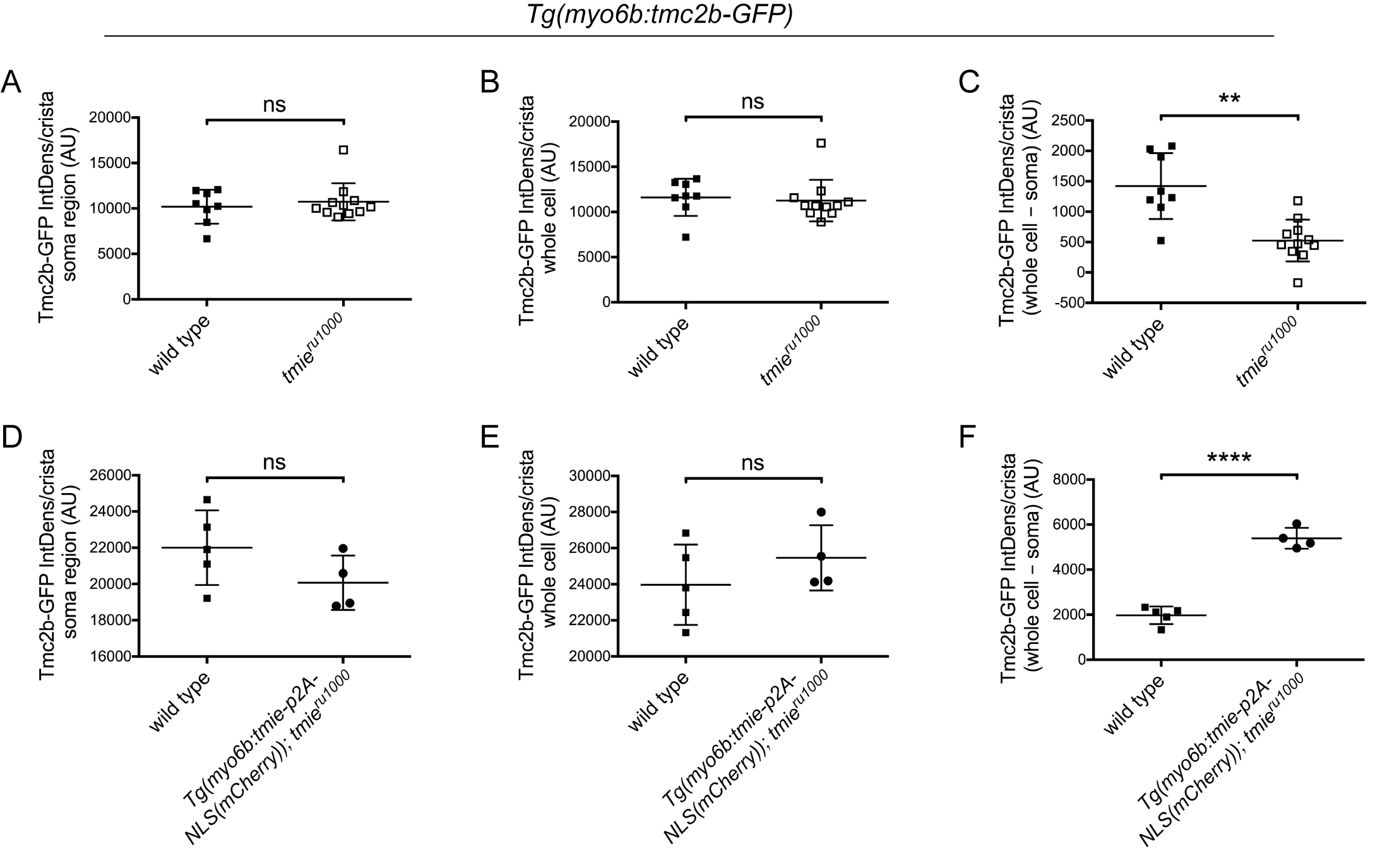

Supplement: S2 Fig — (A-F) Plot of the integrated density of Tmc2b-GFP fluorescence in the ROI of lateral cristae from 4 dpf larvae. ROI in A and D is the soma region, in B and E is the whole hair cell, and in C and F is a subtraction of whole cell fluorescence minus soma fluorescence to roughly determine the relative contribution of bundle signal. Significance was determined by two-tailed unpaired t-test with Welch’s correction, **p < 0.01, ****p < 0.0001. (TIF) [file pgen.1007635.s002.tif]

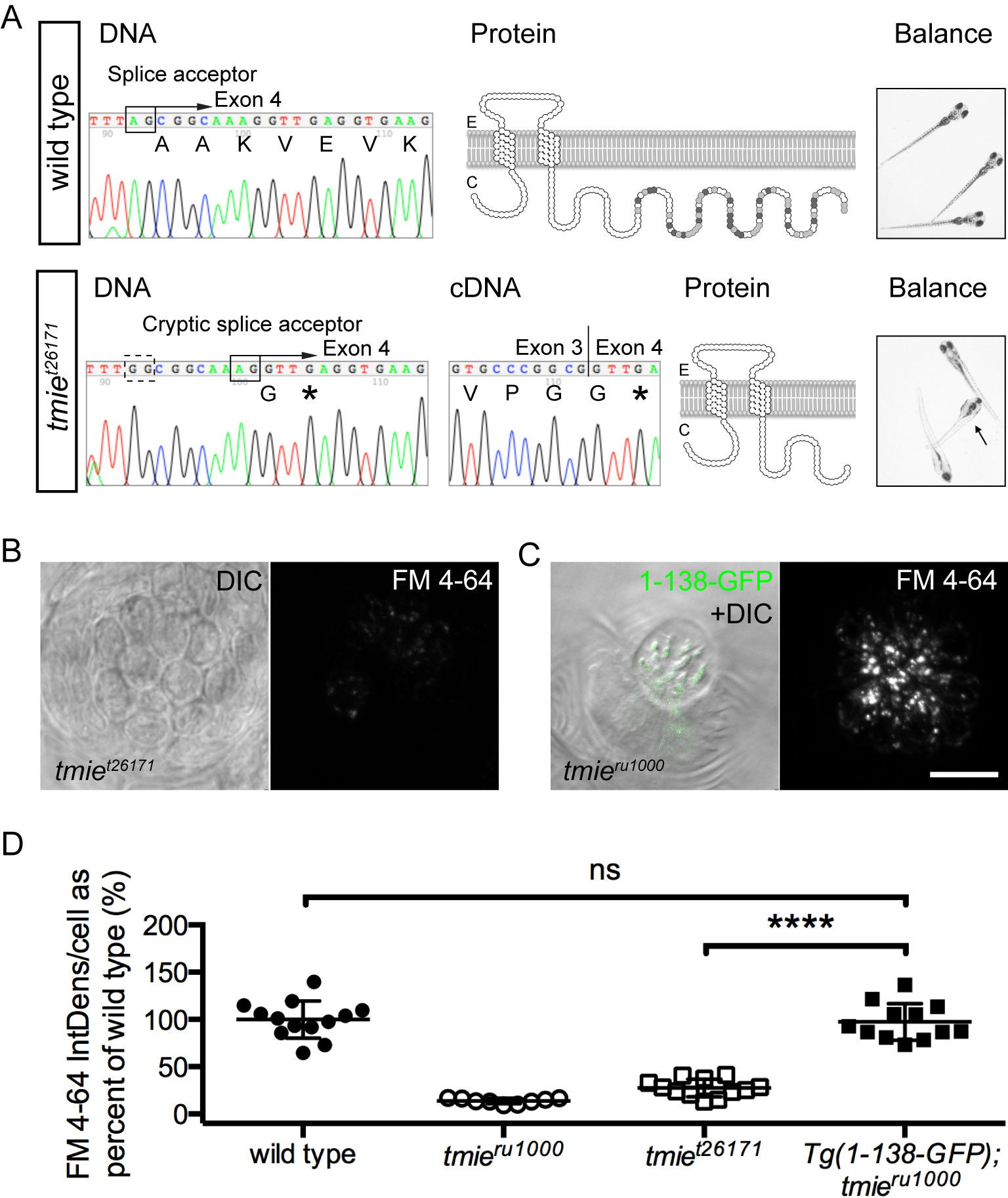

Supplement: S3 Fig — (A) Data for a novel mutant allele of tmie, t26171. DNA: Chromatographs of the DNA sequence of tmie in wild type (above) and tmiet26171 (below) showing the genomic region where the mutation occurs. An arginine is mutated to guanine in the splice acceptor (black box, above) of the final exon of tmie, exon 4. The dashed black box below indicates the mutated original splice acceptor site. Use of a cryptic splice acceptor (black box, below) 8 nucleotides downstream causes a frameshift and an early stop codon (*). cDNA: Chromatograph of the DNA sequence from RT-PCR of tmiet26171 larvae bridging exons 3 and 4. Protein: The predicted protein products, shown here as a two-pass transmembrane protein. The wild type protein has many charged residues (positive in light gray, negative in dark gray) that are lost in tmiet26171. Balance: Photos of wild type and tmiet26171 larvae, taken with a hand-held Canon camera. Arrow points to a larva that is upside-down, displaying a classic vestibular phenotype. (B) Top-down view of a representative neuromast after exposure to FM 4–64, imaged using confocal microscopy. The first panel is a single plane through the soma region while the second panel is a maximum projection of 7 panels through the soma region, beginning at the cuticular plate (as denoted by magenta bracket in Fig 1G). (C) Same as (B) except that the first panel shows the bundle region so that 1-138-GFP can be visualized in bundles (as depicted by dashed green line, Fig 1G). The transgene is driven by the myo6b promoter. (D) Plot of the integrated density of FM fluorescence per cell. We normalized values to the average of wild type siblings. Displayed wild type and tmieru1000 data are from siblings of Tg(1-138-GFP); tmieru1000 and are the same values reported in Fig 6. Data for tmiet26171 is from a separate experiment. Statistical significance determined by one-way ANOVA, ****p<0.0001. Scale bar is 10μm. (TIF) [file pgen.1007635.s003.tif]

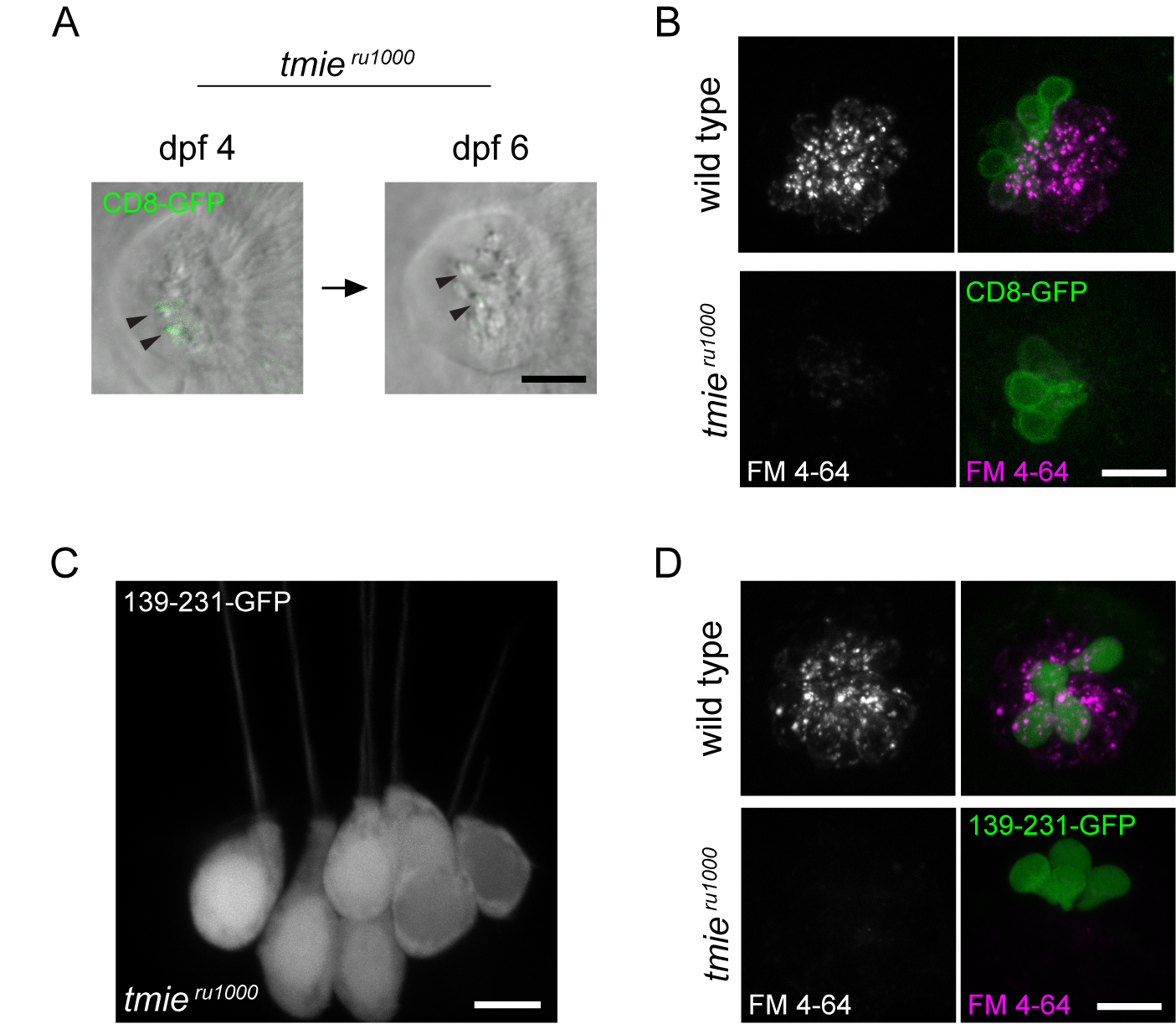

Supplement: S4 Fig — All images were captured using confocal microscopy. (A) Stereocilia of a neuromast viewed from above. The same neuromast was imaged at 4 dpf and 6 dpf. In hair cells expressing CD8-GFP, signal was initially detected in immature bundles, but this expression was only detectable in soma by dpf 6 as the cells matured (n = 10 cells). (B) Maximum projection of neuromasts viewed from above; left panel shows only FM 4–64 while right panel adds CD8-GFP. No rescue of FM 4–64 labeling was observed in tmieru1000 hair cells expressing CD8-GFP (n = 40 cells). (C) Maximum projection of the posterior crista in a tmieru1000 larva with some hair cells expressing 139-231-GFP, which fills the cell (n = 43 cells). (D) Same as B except the transgene being expressed is 139-231-GFP. No rescue of FM 4–64 labeling was observed in tmieru1000 hair cells expressing 139-231-GFP (n = 33 cells). Scale bars in A and C are 5μm, in B and D are 10μm. (TIF) [file pgen.1007635.s004.tif]

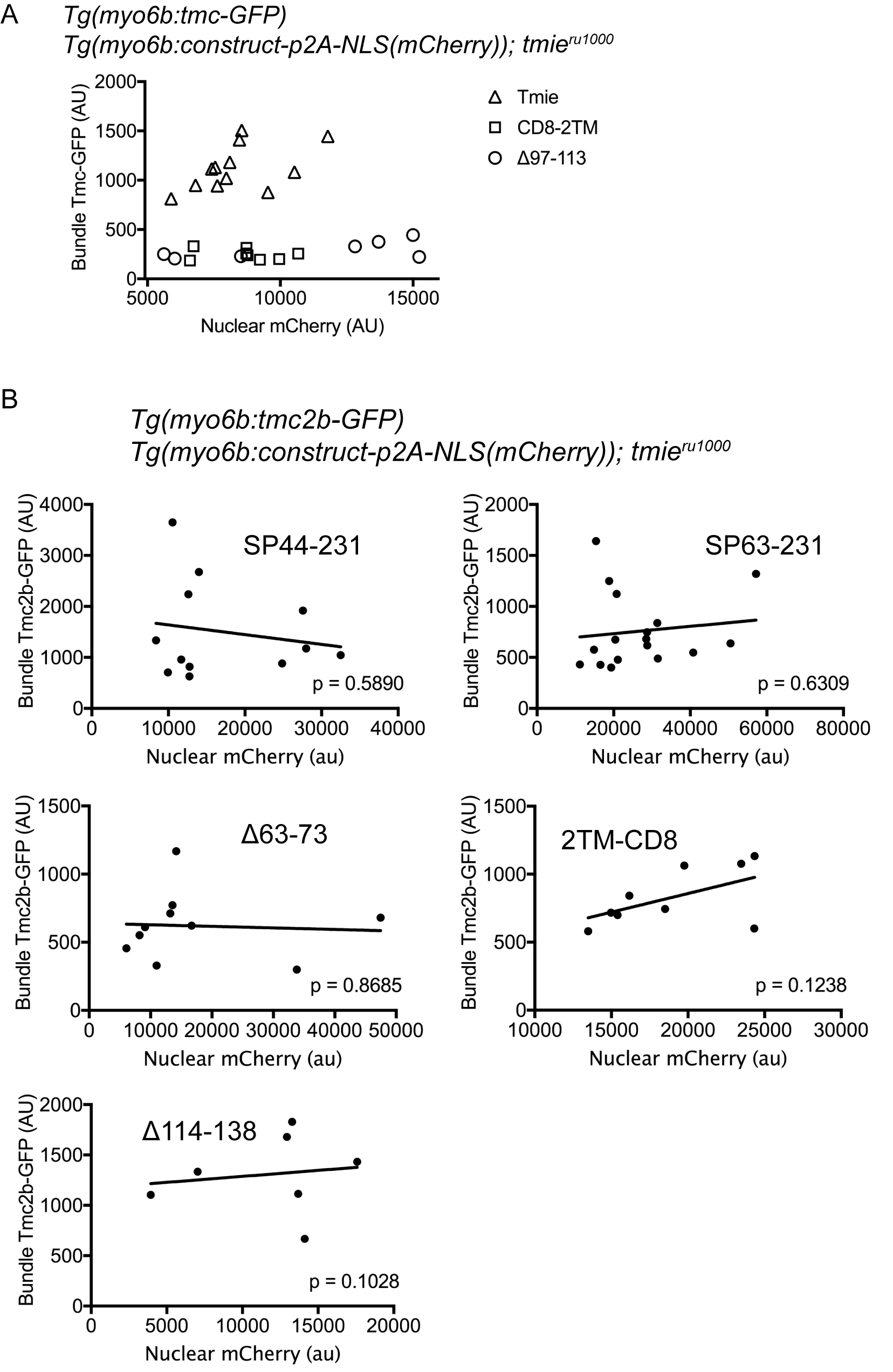

Supplement: S5 Fig — XY plots of the integrated density of nuclear mCherry fluorescence vs the integrated density of GFP-tagged Tmc fluorescence in the bundle region of lateral cristae. We examined 4 dpf larvae. (A) Bundle values for constructs CD8-2TM and Δ97–113 are the same as those reported in Fig 8H using co-expression with Tmc2b-GFP. Bundle values for the full-length Tmie construct are the same as those reported in Fig 4C using co-expression with Tmc1-GFP. (B) Bundle values are the same as those reported in Fig 8 using co-expression of each individual tmie construct with Tmc2b-GFP. We performed linear regressions to generate p-values. (TIF) [file pgen.1007635.s005.tif]

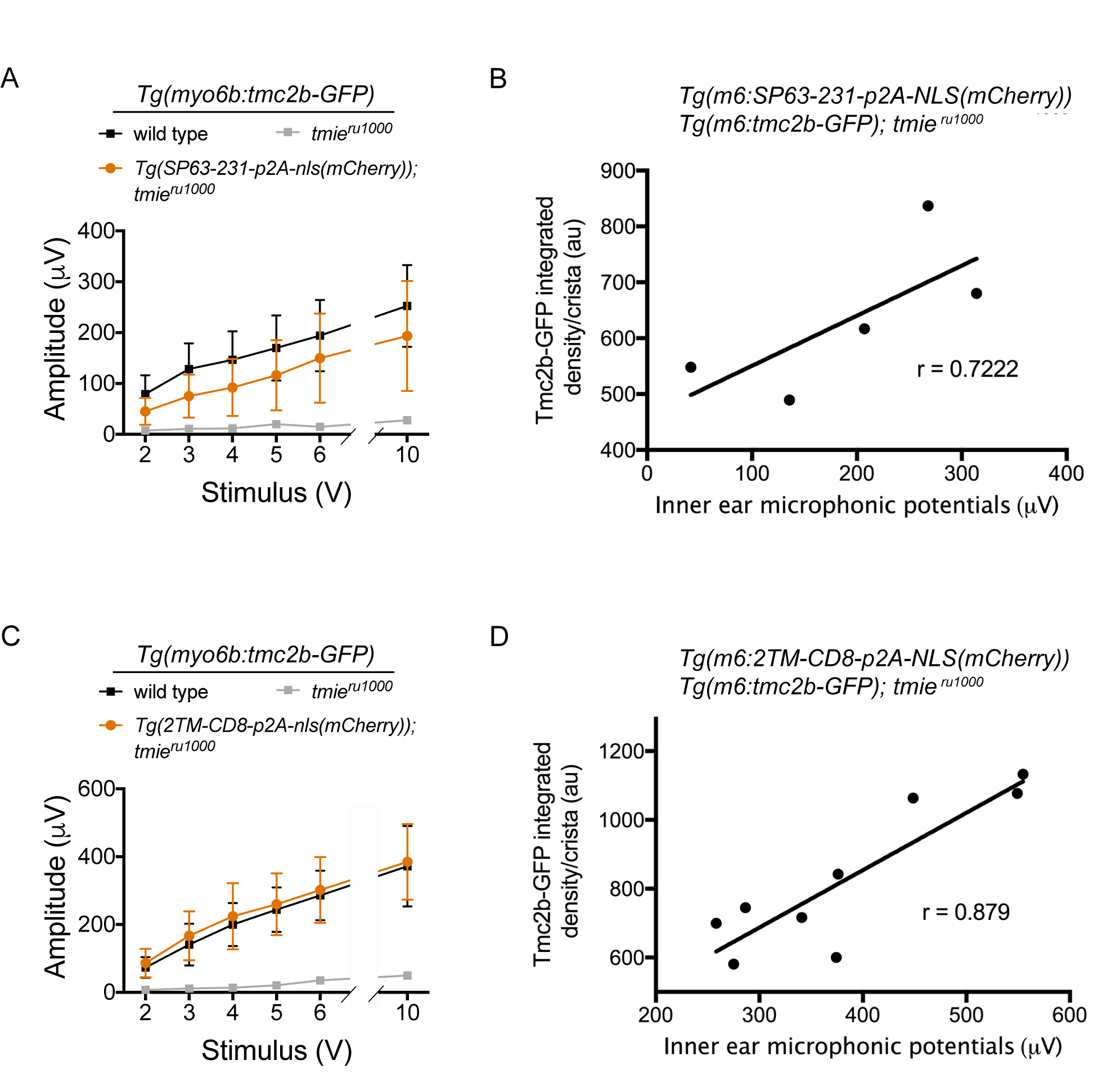

Supplement: S6 Fig — (A) Mean amplitude of the response peak ± SD as a function of the stimulus intensity of the driver voltage, as described in Fig 7B. (B) XY plot of the amplitude of microphonic response vs the integrated density of Tmc2b-GFP fluorescence in the ROI. A 10V step stimulus was used to evoke microphonic potentials. The line is a linear regression with a Pearson r = 0.7222, p = 0.1682. (C) Same as A except with the 2TM-CD8 construct. (D) Same as B except with the 2TM-CD8 construct, r = 0.879, p = 0.0018. Significance determined by Pearson correlation coefficient. Measurements were from 4 dpf larvae; we used lateral cristae for imaging of Tmc2b-GFP. (TIF) [file pgen.1007635.s006.tif]

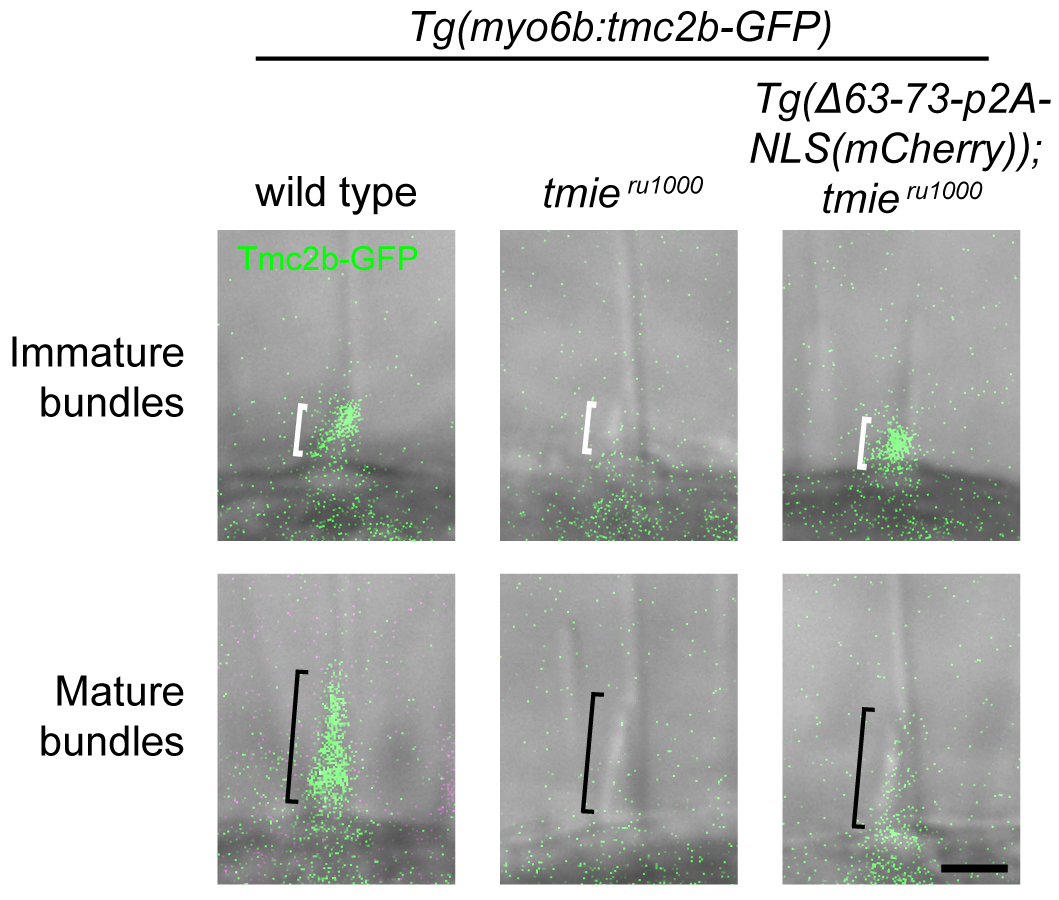

Supplement: S7 Fig — Confocal images of single hair bundles from cells expressing transgenic Tmc2b-GFP driven by the myo6b promoter. Brackets show the stereocilia bundle, which is shorter in immature hair cells (white brackets) and longer in mature ones (black brackets). We examined lateral cristae from 4 dpf larvae. Scale bar is 2μm. (TIF) [file pgen.1007635.s007.tif]

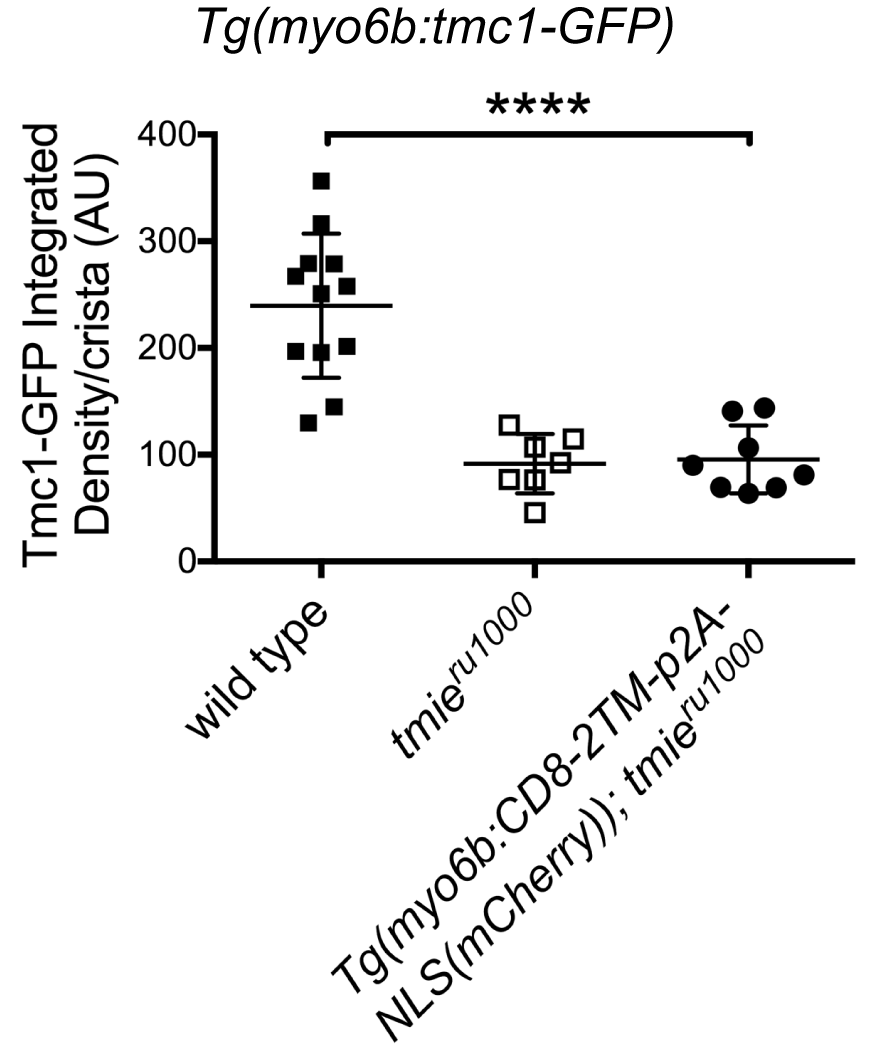

Supplement: S8 Fig — Plot of the integrated density of Tmc1-GFP fluorescence in the ROI, expressed as arbitrary units. We examined lateral cristae from 4 dpf larvae. Significance was determined by one-way ANOVA, n ≥ 7, ****p < 0.0001. (TIF) [file pgen.1007635.s008.tif]
